# The Effect of Online Hemodiafiltration on Infections: Results from the CONvective TRAnsport STudy

**DOI:** 10.1371/journal.pone.0135908

**Published:** 2015-08-19

**Authors:** Claire H. den Hoedt, Muriel P. C. Grooteman, Michiel L. Bots, Peter J. Blankestijn, Ingeborg van der Tweel, Neelke C. van der Weerd, E. Lars Penne, Albert H. A. Mazairac, Renée Levesque, Piet M. ter Wee, Menso J. Nubé, Marinus A. van den Dorpel, CONTRAST investigators

**Affiliations:** 1 Department of Internal Medicine, Maasstad Hospital, Rotterdam, The Netherlands; 2 Department of Nephrology, University Medical Center Utrecht, Utrecht, The Netherlands; 3 Department of Nephrology, VU Medical Center, Amsterdam, The Netherlands; 4 Institute for Cardiovascular Research VU Medical Center (ICaR-VU), VU Medical Center, Amsterdam, The Netherlands; 5 Julius Center for Health Sciences and Primary Care, University Medical Center Utrecht, Utrecht, The Netherlands; 6 Academic Medical Center, Amsterdam, The Netherlands; 7 Medical Center Alkmaar, Alkmaar, The Netherlands; 8 Centre Hospitalier de l’Université de Montréal, St. Luc Hospital, Montréal, Canada; Cardiff University, UNITED KINGDOM

## Abstract

**Background:**

Hemodialysis (HD) patients have a high risk of infections. The uremic milieu has a negative impact on several immune responses. Online hemodiafiltration (HDF) may reduce the risk of infections by ameliorating the uremic milieu through enhanced clearance of middle molecules. Since there are few data on infectious outcomes in HDF, we compared the effects of HDF with low-flux HD on the incidence and type of infections.

**Patients and Methods:**

We used data of the 714 HD patients (age 64 ±14, 62% men, 25% Diabetes Mellitus, 7% catheters) participating in the CONvective TRAnsport STudy (CONTRAST), a randomized controlled trial evaluating the effect of HDF as compared to low-flux HD. The events were adjudicated by an independent event committee. The risk of infectious events was compared with Cox regression for repeated events and Cox proportional hazard models. The distributions of types of infection were compared between the groups.

**Results:**

Thirty one percent of the patients suffered from one or more infections leading to hospitalization during the study (median follow-up 1.96 years). The risk for infections during the entire follow-up did not differ significantly between treatment arms (HDF 198 and HD 169 infections in 800 and 798 person-years respectively, hazard ratio HDF vs. HD 1.09 (0.88–1.34), *P* = 0.42. No difference was found in the occurrence of the first infectious event (either fatal, non-fatal or type specific). Of all infections, respiratory infections (25% in HDF, 28% in HD) were most common, followed by skin/musculoskeletal infections (21% in HDF, 13% in HD).

**Conclusions:**

HDF as compared to HD did not result in a reduced risk of infections, larger studies are needed to confirm our findings.

**Trial Registration:**

ClinicalTrials.gov NCT00205556

## Introduction

Infections are an important cause for morbidity and mortality in hemodialysis (HD) patients [1]. The uremic environment affects both the innate and the adaptive immune response by dysfunction of neutrophilic polymorphonuclear leukocytes [2] and monocytes, a depletion of dendritic cells, naïve and central memory T cells and B cells [3]. In addition, frequent damaging of the skin in case of a graft or arterio-venous fistula or the presence of a central venous catheter facilitates the entry of micro-organisms into the body. Patients with a catheter as a vascular access have the largest infectious risk, followed by patients with grafts [4–6]. Several patient related factors contribute to the susceptibility to infection, including older age, hypo-albuminemia, co-morbidity, personal hygiene and underlying chronic infectious disease [5,7,8].

Theoretically, amelioration of the uremic milieu by improving clearance of middle molecules by convection might reduce this risk of infections. The HEMO study however, comparing low-flux with high-flux HD, did not show a decrease in the risk of infections for patients treated with high flux membranes [5]. Online hemodiafiltration (HDF), a therapy that achieves markedly better clearance of middle molecules than high-flux HD, might reduce the incidence of infections. However, removal of middle molecules might also lead to a depletion of immune effector molecules, pro-inflammatory cytokines, or other mediators relevant for immune function. In addition, despite tight monitoring and quality control, the infusion of large amounts of substitution fluid, when contaminated, might impose a larger risk for infections. So far the effect of HDF on the incidence of infectious episodes as compared to low-flux HD has hardly been studied. Therefore, we performed a secondary analysis of data of the CONvective TRAnsport STudy (CONTRAST) to evaluate the effect of HDF as compared to low-flux HD on the incidence and causes of infection related hospitalizations and mortality.

## Methods

### General methods

The CONvective TRAnsport STudy (CONTRAST) (ISRCTN38365125, NCT00205556) is a randomized controlled trial, conducted in twenty-nine dialysis centers in The Netherlands (n = 26), Canada (n = 2), and Norway (n = 1), that compared the effects of low-flux HD and online post-dilution HDF on all-cause mortality and cardiovascular morbidity and mortality [9,10]. Patients were eligible if treated with HD 2 or 3 times a week, for at least 2 months, with a single pool Kt/V_urea_ ≥ 1.2. Exclusion criteria were age < 18 years, treatment by HDF or high-flux HD in the 6 months preceding randomization, severe incompliance defined as non-adherence to the dialysis prescription, a life expectancy < 3 months due to causes other than kidney disease, and participation in another clinical intervention study evaluating cardiovascular outcome. All patients were randomized centrally by a computer-based randomization service (Julius Center University Medical Center, Utrecht, The Netherlands) into a 1:1 ratio for treatment with online hemodiafiltration or continuation of low-flux hemodialysis, stratified per participating center (permuted blocks). Because of the nature of the intervention, it was not possible to blind the patients, the local study nurses, or the investigators to the treatment assignment. The laboratory samples were measured in routine clinical care; hence, personnel were unaware of treatment assignment. The event adjudication committee was blinded to the treatment assignment. Patients were enrolled from June 2004 until January 1^st^ 2010 by nephrologists and research nurses in participating centers. Follow-up ended December 31^st^ 2010. The study was conducted in accordance with the Declaration of Helsinki and centrally approved by the Medical Ethical committee VU University medical Center, Amsterdam, The Netherlands, for all the participating hospitals on the 31^st^ of July 2003, an amendment was approved on 28^th^ of June 2007. Local approval was obtained from the Medical Ethical Committees of all participating centers, which are listed in the acknowledgements. The authors confirm that all ongoing and related trials for this intervention are registered. Written informed consent was obtained from all patients prior to randomization. The study was registrated in the trial registries after the first patients were enrolled, immediately by the time the investigators knew trial registration was obligatory. The assessment of difference in risk of infections between dialysis modalities was one of the predefined secondary outcomes [10].

### Dialysis procedures

Treatment times were fixed during follow-up in both treatment arms, unless single pool Kt/V_urea_ was below 1.2. Online HDF was performed in the post-dilution mode; target volume was 6 L/h. For HDF, synthetic high-flux dialyzers were used (FX80: 24%, FX100: 12% and Optiflux F200NR: 9% [Fresenius Medical Care, Bad Homburg, Germany]; Polyflux 170H: 20% and Polyflux 210H: 30% [Gambro AB, Lund, Sweden] or other dialyzers: 4%, based on data of 3 month visit). HD patients were treated with synthetic low-flux dialyzers (F6HPS: 4%, F8HPS: 46% and Optiflux 18NR: 11% [Fresenius]; Polyflux 14L: 4%, Polyflux 17L: 25% and Polyflux 21L: 4% [Gambro], or other: 6%, based on data of 3 month visit). All patients were treated with ultra pure dialysis fluids, defined as less than 0.1 colony forming units per mL and less than 0.03 endotoxin units per mL. Routine patient care was performed according to national and international Quality of Care Guidelines.

### Data collection

At baseline standardized forms were used to collect demographical, clinical and laboratory data. Type of vascular access, duration of dialysis (dialysis vintage), and medical history (presence of diabetes mellitus (DM) and previous cardiovascular disease (CVD)), were also recorded. CVD was defined as a history of angina pectoris, myocardial infarction, prior coronary revascularization, stroke or transient ischemic attack and/or peripheral vascular disease. Dialysis vintage was determined as the sum of time patients were treated with HD or peritoneal dialysis (PD) before inclusion in CONTRAST.

At each three monthly visit, data on clinical events (including infections), dialysis treatment, medication, and laboratory values were recorded. Infectious events were registered for all patients before drop out due to transplantation, switch to PD, move to another clinic or stop for other reasons. All infectious events were adjudicated by an independent Endpoint Adjudication Committee, whose members reviewed source documentation and were not aware of the treatment assignments. Infections were adjudicated as categorized definite or probable when patients were admitted to the hospital with a clinical picture of an infection, and with laboratory results suggesting an infection (leukocytosis, elevated CRP) or when infection was proven by culture. Infections were also adjudicated when these occurred during an admission for another cause. Only definite and probable infections were used for this analysis, possible infections (when a patient was admitted with only a clinical picture of infection, but without elevated inflammation parameters or a positive culture result) were considered as no infections. A report of two or more infections within a timeframe of 14 days was counted as one infection. If the second infection was fatal, that infection was used in the analysis. Infections were grouped as graft or fistula infection, catheter sepsis, sepsis, respiratory, urinary and ‘other infections’. Those that were categorized as ‘other infection’ have been subdivided into gastro-intestinal, skin/musculoskeletal, cardiac and miscellaneous infections retrospectively. Written diagnoses from the adjudication committee were used for this categorization.

In HDF patients, infusion volumes (liters per treatment) were reported as the mean value of three consecutive treatment sessions. Convection volumes (liters per treatment) were calculated by the sum of the intradialytic weight loss and the substitution volume. Patients with a urinary production of less than 100 mL per day were considered anuric.

Routine laboratory values were measured in the different participating hospitals using standard techniques.

### Outcome

The primary study outcome was the risk of hospitalizations due to infection during the follow-up period. The secondary outcomes were mortality due to an infectious cause, the risk for the 1^st^ fatal or non-fatal infection and the risk for 1^st^ cause specific infection. Furthermore we studied the distribution of cause specific infections on the event level (enabling the analysis of more than one infectious event per patient).

All infectious events that occurred after randomisation up to censoring were used in our analyses. Censoring could be due to death, due to end of study or due to dropping out. Dropping out means that a participant either stopped because of a renal transplantation (n = 151), switch to PD (n = 11), transfer to another hospital which did not participate in CONTRAST (n = 24) or due to other reasons (n = 53).

### Data analysis

Data were reported as means with standard deviations, medians with interquartile (IQR) ranges, or proportions when appropriate.

The difference in risk for hospitalization due to infections between patients treated with HDF and HD during the follow-up was evaluated with a Cox proportional hazards model for repeated events, which is a Prentice-Williams-Peterson (PWP) conditional model [11]. In this model patients have a number of follow-up periods, depending on the number of events. The model takes intra-patient risk for infections into account. Furthermore we added interaction-terms to this model to explore whether the difference in risk between HDF and HD treated patients was different for certain predefined subgroups, notable age, sex, presence of CVD, presence of DM, presence of RKF and dialysis vintage. Furthermore, we explored whether the risk of infections was affected by the magnitude of the delivered convection volumes during the trial, with using the HD group as a reference group. In this last model adjustments were made for determinants of convection volume and mortality. Finally, since the distribution of vascular access type was somewhat different between patients from the Netherlands and Canada, we evaluated if there was an interaction between the treatment and the country of residence and performed additional analyses on these countries separately, with an adjustment for vascular access type.

The difference in risk for 1^st^ infectious events (fatal- and nonfatal and cause specific infections) between HDF and HD was analyzed with Cox proportional hazard models. The distribution of infectious events from different causes was compared on the event level. The analyses were conducted in SPSS software (version 18.0; SPSS Inc. Headquarters, Chicago, Illinois, US) and in R (version 2.9.2; 2009 The R Foundation for Statistical Computing).

## Results

**Fig 1:**
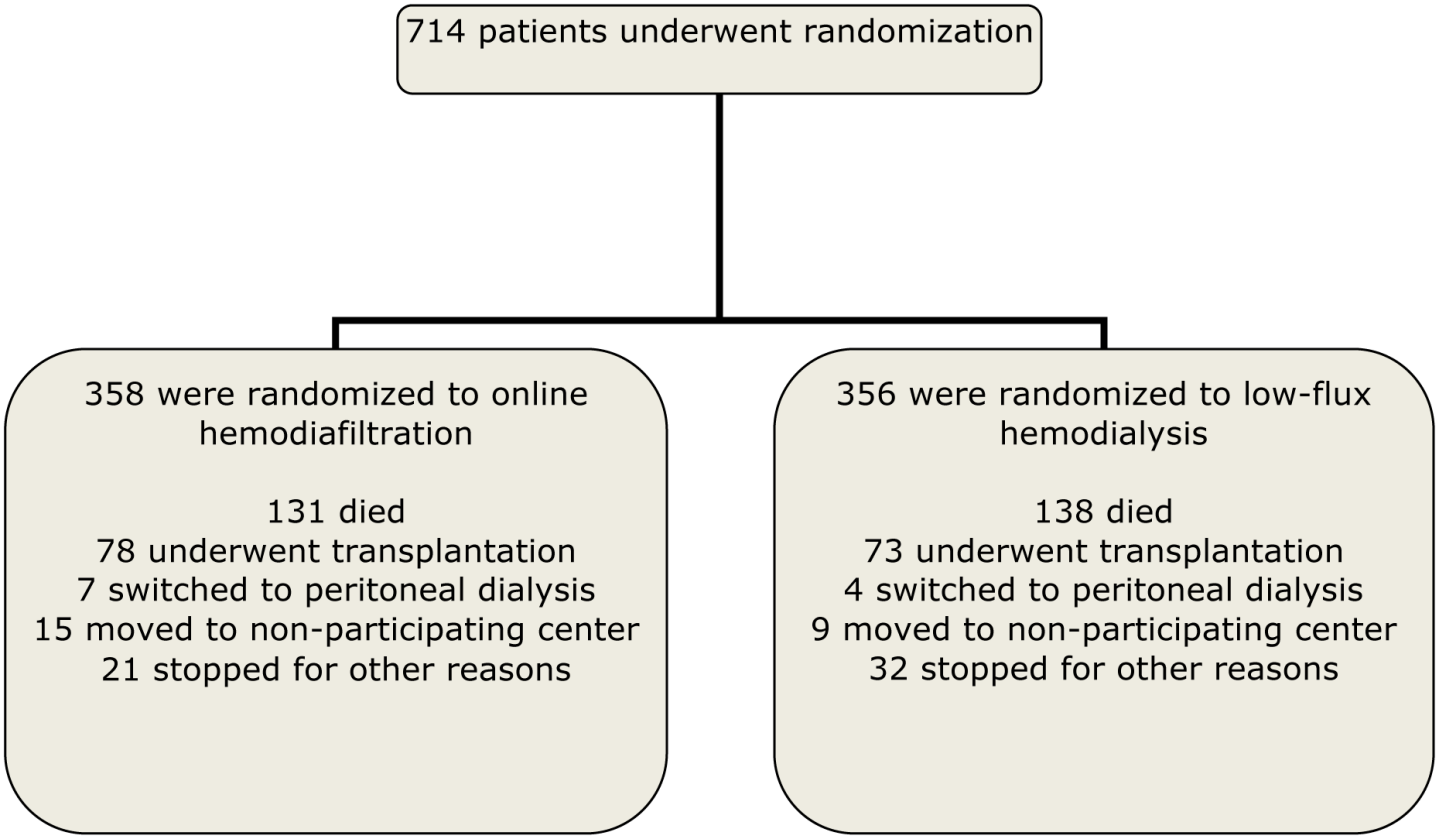
Enrolment, randomization, and follow-up of study participants. For infections, all patients were followed until drop out, death or the end of the study.

Patient characteristics at baseline are shown in Table 1. The participant flow chart is shown in Fig 1. Thirty one percent of the patients suffered from one or more infections leading to hospitalization during the study (median follow-up 1.96 years). The treatment effect on risk of infectious events is depicted in Table 2. HDF did not reduce the risk of infections: hazard ratio (HR) HDF versus HD 1.09 (0.88–1.34), *P* = 0.42. In addition, no statistically significant differences were found between the two treatment arms for the first occurring infection (Table 2).

**Table 1:** Baseline characteristics of the patients.

Variable	HDF(n = 358)	HD(n = 356)
Age (year)	64.1±14.0	64.0±13.4
Male sex—no. (%)	214 (60)	231 (65)
Region		
Netherlands-no. (%)	300 (84)	297 (83)
Canada- no. (%)	51 (14)	51(14)
Norway- no. (%)	7 (2)	8 (2)
History of cardiovascular disease—no. (%)	151 (42)	162 (46)
Diabetes mellitus—no. (%)	92 (26)	78 (22)
Body mass index after dialysis—kg/m^2^	25.2±5.0	25.6±4.6
Dialysis vintage (year)		
-Median (inter-quartile range)	1.8 (1.0–3.7)	2.1 (1.0–4.0)
Systolic blood pressure—mmHg	147±21	148±22
Diastolic blood pressure-mmHg	75±12	76±12
Vascular access		
Arteriovenous fistula- no. (%)	279 (78)	288 (81)
Graft- no. (%)	57 (16)	43 (12)
Central catheter- no. (%)	22 (6)	25 (7)
Number of treatments/week		
-3- no. (%)	332 (93)	338 (95)
-2- no. (%)	26 (7)	18 (5)
Duration of a dialysis session—min	226±26	227±22
Blood flow—mL/min	302±39	299±41
Dialysis single pool Kt/V_urea_	1.41±0.24.	1.38±0.19
Residual kidney function no.(%)*	186 (52)	190 (53)
Estimated glomerular filtration rate		
-ml/min/1.73m^2^; median (inter-quartile range)	0.32 (0–3.30)	0.30 (0–3.35)
Hemoglobin—mmol/L	7.4±0.82	7.3±0.73
Phosphorus—mmol/L	1.65±0.51	1.63±0.47
Beta-2-microglobulin—mg/L	30.7±14.3	32.3±13.6
Albumin—g/L^	40.2±3.8	40.6±3.9
Creatinine–μmol/L pre-dialysis	842±260	879±250

Values are means ±SD, median (interquartile range) or number (percentage).

HDF = online hemodiafiltration; HD = hemodialysis;

^~^pre-dialysis

*residual kidney function if diuresis >100 ml/24h

^^^albumin concentrations measured with the bromcresolpurple method have been converted to the bromcresolgreen method

To convert hemoglobin in mmol/L to g/dL divide by 0.62; phosphorus in mmol/L to mg/dL, divide by 0.323; albumin in g/L to g/dL, divide by 10; creatinine in μmol/L to mg/dL divide by 88.4

**Table 2:** The risk for infectious events in patients treated with HD and HDF

	HDF (n = 358)		HD (n = 356)			
Event	No. of events	Person-years of FU	No. of events	Person-years of FU	HR HDF vs HD (95%CI)	*P*
All infections	198	800	169	798	1.09 ^¶^	0.42
					(0.88–1.34)	
Fatal infection*	23	800	28	798	0.85 ^	0.56
					(0.49–1.47)	
1^st^ Graft- or fistula infection	11	787	11	788	0.99 ^	0.98
					(0.43–2.29)	
1^st^ Catheter sepsis	14	787	8	781	1.77 ^	0.20
					(0.74–4.22)	
1^st^ Sepsis	17	790	7	794	2.40 ^	0.05
					(1.00–5.79)	
1^st^ Urinary tract infection	10	792	12	781	0.82 ^	0.64
					(0.35–1.89)	
1^st^ Respiratory infection	37	744	38	756	0.98 ^	0.98
					(0.62–1.53)	
1^st^ Other infection	52	735	38	755	1.40 ^	0.11
					(0.92–2.13)	
1^st^ non-fatal or fatal infection	118	652	106	681	1.16 ^	0.27
					(0.89–1.51)	

HDF = Online hemodiafiltration; HD = Low-flux hemodialysis; No. = number; FU = follow-up; HR = hazard ratio

^¶^ analyzed with Cox for repeated events

^^^obtained through unadjusted Cox proportional hazards models, time to (first) infectious event

*On treatment analysis, so infectious death on treatment of HD or HDF or within 28 days after censoring due to transplantation, switch to PD, move to other clinical or stop for other reasons

No difference in the effect of HDF was found in subgroups of age (below or above the median of 67 yrs, *P value for the interaction term* = 0.44), sex (*P* = 0.49), DM (present/absent, *P* = 0.64), previous CVD (*P* = 0.14), RKF (present/absent, *P* = 0.42) and dialysis vintage (below or above the median of 2 yrs, *P* = 0.20), or country of residence (*P value for the interaction term* = 0.15). We found a trend towards a different effect of HDF in patients with high or low serum albumin levels (*P for interaction* = 0.05). The HR (HDF vs. HD) for infections was 1.31 (0.96–1.79, *P* = 0.09) in patients with a baseline serum albumin above 40.5 g/L (median), and was 0.86 (0.65–1.13, *P* = 0.29) in patients with a baseline serum albumin below the median.

Compared to HD, the incidence of infections did not differ across tertiles of convection. Lowest convection volume tertile HR 1.10 (0.84–1.45), *P* = 0.49, middle convection tertile HR 1.13 (0.85–1.50), *P* = 0.41, highest convection tertile HR 0.88 (0.64–1.20), *P* = 0.42 (all versus HD and adjusted for country of origin). This result remained unaltered after adjustment for age, sex, CVD, DM, hematocrit, serum albumin, treatment time, blood flow rate, vascular access and site.

Of all infections, respiratory infections (25% in HDF, 28% in HD) were most common, followed by skin/musculoskeletal infections (21% in HDF, 13% in HD) (Tables 3 and 4).

**Table 3:** Total number of infectious events by cause

	HDF			HD		
Event	Total no. of events	In n patients	% of all infections	Total no. of events	In n patients	% of all infections
			(95% CI)			(95% CI)
Fatal infection*	23	23	11.6	28	28	16.6
			(7.1–16.1)			(11.0–22.2)
Graft- or fistula infection	11	11	5.6	12	11	7.1
			(2.4–8.7)			(3.2–11.0)
Catheter sepsis	16	14	8.1	8	8	4.7
			(4.3–11.9)			(1.5–7.9)
Sepsis	18	17	9.1	7	7	4.1
			(5.1–13.1)			(1.1–7.1)
Urinary tract infection	12	10	6.1	15	12	8.9
			(2.7–9.4)			(4.6–13.2)
Respiratory infection	49	37	24.7	47	38	27.8
			(18.7–30.8)			(21.1–34.6)
Other infection	69	52	34.8	52	38	30.8
			(28.2–41.5)			(23.8–37.7)
Total no. of infections	198	118	100	169	106	100

HDF = Online hemodiafiltration; HD = Low-flux hemodialysis; no. = number

*On treatment analysis, so infectious death on treatment of HD or HDF or within 28 days after censoring due to transplantation, switch to PD, move to other clinic or stop for other reasons

Note: one patient may have more than one infectious event

**Table 4:** Distribution of other infections in online hemodiafiltration and hemodialysis

	HDF		HD	
	No.	% of all infections	No.	% of all infections
Gastro-intestinal	17	8.6 (4.7–12.5)	13	7.7 (3.7–11.7)
Skin/musculoskeletal	42	21.2 (15.5–26.9)	22	13.0 (7.9–18.1)
Cardiac	2	1.0 (0–2.4)	1	0.6 (0–1.7)
Miscellaneous	8	4.0 (1.3–6.8)	16	9.5 (5.1–13.9)
Total	69		52	

HDF = online hemodiafiltration; HD = hemodialysis; No. number

Table 5 shows that the distribution of access types was different between patients treated in the Netherlands and in Canada. The risk for infections was neither changed by HDF in the Netherlands (HR 1.15 (0.91–1.45, *P* = 0.23) nor in Canada (HR 0.93 (0.55–1.56, *P* = 0.79). Since the proportion of patients treated with grafts in the Netherlands was larger in the HDF group, we adjusted for access type at baseline. The HR’s for the Netherlands and for Canada remained however comparable (1.13 (0.90–1.43, P = 0.29) and 0.93 (0.55–1.56, P = 0.78) respectively.

**Table 5:** Vascular access types at baseline in the Netherlands versus Canada.

	HDF		HD	
	**The Netherlands**			
**Vascular access**	**No.**	**%**	**No.**	**%**
AV-fistula	245	82	256	86
Graft	49	16	34	11
CVC	4	1	5	2
	**Canada**			
AV-fistula	29	57	28	55
Graft	7	14	5	10
CVC	15	29	18	35

HDF = online hemodiafiltration; HD = hemodialysis; No. number; AV = arterio-venous; CVC = central venous catheter

## Discussion

In this large randomized controlled trial we found that 31% of the participants suffered from one or more infections leading to hospitalization during the study (median follow-up 1.96 years). Our results suggest that treatment with HDF does not reduce the risk of mortality and hospitalization due to infections as compared to HD. The most common infections in both treatment arms were respiratory infections and skin/musculoskeletal infections.

To our knowledge, this is the first study investigating the risk of infections in patients treated with HDF as compared to patients treated with low-flux HD. Our data indicate that alteration of the uremic milieu by convective clearance does not reduce the risk of infections. Our data are in line with the results of the HEMO study, which showed that high flux HD did not decrease the risk of infectious outcomes [5]. Also in the Turkish HDF study no difference in infectious related mortality was found between HDF and high-flux HD patients[12]. However, they only reported mortality from infections[12]. Our results are different from the results of the Spanish ESHOL study in which a reduced mortality by infections was found in the online HDF group as compared to the high-flux HD group[13]. However, they neither showed a difference in the number of infectious related hospitalizations between patients treated with HDF and HD. They did not report categories of infection[13].

In our study we found a trend towards a higher risk of sepsis in HDF patients. However, it may be a chance finding, since the number of patients in whom a sepsis or catheter sepsis occurred was small. Also, if we would take multiple testing into account, the association would be far from statistically significant. We do not have a theoretical explanation for the trend towards an increased number of sepsis in the HDF group. We neither have an explanation for a potentially different effect of HDF in patients with an albumin below or above the median, this might be a chance finding as well.

The impact of various dialysis modalities on immune function is speculative. There is no evidence that alteration of dialysis efficiency or improving clearance of middle molecules from the blood would have a beneficial effect on immune processes on the tissue level. HDF might enhance the clearance of deleterious molecules such as complement factor D, granulocyte inhibitory protein II (GIP II) and immunoglobulin free light chains, which have been shown to have an in vitro depressant effect on degranulation, chemotaxis and phagocytosis of mainly polymorphonuclear leukocytes [14]. Alternatively, HDF may also enhance the clearance of immune-active molecules such as cytokines, or other unknown molecules essential for immune function. The limited data on clearance of specific toxins by HDF and the in vitro and in vivo function of these toxins makes it very difficult to explain clinical outcomes. In addition, despite tight monitoring and quality control, the infusion of large amounts of substitution fluid, when contaminated, might impose a larger risk for infections. However we showed in a previous study that microbiological cultures of the substitution fluid were negative in 98% of 193 tested samples and endotoxin levels were below the reference quality level in 98% of 177 tested samples [15].

The groups of respiratory infections (25–28%) and skin/musculoskeletal infections (13–21%) were the most common infections in our study. These infections occurred more often in the same patients as well. In the HEMO study the contribution of respiratory disease as the cause for an infection related hospitalization was 22% as well [5]. Infections from cardiac, peripheral vascular disease, DM, hepatobiliary disease, musculoskeletal and gastrointestinal causes, which were included in our category ‘other infections’, also contributed to 31% of infection related hospitalizations [5]. The contribution of septicaemia and bacteremia and infections due to vascular access complications was larger than in our study, despite a comparable use of catheters in only 8% of patients [5]. Considering respiratory infections, a study from the United States (US) retrospectively linked Medicare claims to Dialysis Morbidity and Mortality Study data sets and reported that over an average follow-up of 3.3 years 28.9% of the study population was hospitalized with a pneumonia [16], which was 11% in a median follow-up of 1.96 years in our study. Another study from the US also based on claim data of Medicare reported a septicaemia rate of 17.5 per 100 patient years in HD patients in 1999, which is probably due to a high use of catheters [17]. The risk of non vascular access related infections, septicaemia related hospitalizations and infections of vascular access reported from an analysis of 5 European DOPPS countries was comparable to our study [18]. It remains however difficult to compare studies from different continents and data that are collected retrospectively or prospectively.

The strengths of our study are the randomized design and the prospective collection of the data on infectious events, including the categories of infection. Furthermore, all infectious events were adjudicated and all infections during the on treatment follow-up were taken into account in the data-analyses. A limitation is the fact that this study was powered on mortality and not on infections, so the study is underpowered to detect smaller, clinically relevant, differences. So a beneficial or harmful effect of treatment with HDF cannot be ruled out completely based on our findings. As the 95% of the HR (1.09) was 0.88–1.34, the effect of online HDF may vary between a 12% reduction and a 34% increase in hospitalization for infections, both extremes being clinically relevant. Most important in this superiority trial, a small beneficial effect for HDF can still exist. Furthermore the lack of cause specific registration of fatal infections, the retrospective categorization of the category ‘other infections’ and the generalizability to non-European countries, with a different composition of vascular accesses might be limitations.

More and larger studies are needed to confirm our findings. These studies should be powered on the incidence on infections, to rule out that the lack of effect we found, is not due to a lack of power. These studies should be designed as inferiority trials, to explore the trend towards more sepsis in the HDF group. The FINESSE trial, comparing high-flux HD with HDF, will investigate episodes of septicaemia as a secondary safety outcome [19]. Furthermore, data from the HDF Pooling Project could confirm our findings [20].

In conclusion, as compared to HD, our data suggest that treatment with HDF does not reduce the risk of infectious episodes in patients with end stage renal disease.

## Supporting Information

S1 CONSORT Checklist CONSORT 2010 checklist. (DOC)

S1 Protocol Study Protocol. (PDF)
