# Supplementary material for: The Effect of Online Hemodiafiltration on Infections: Results from the CONvective TRAnsport STudy
Source: PLoS One. 2015 Aug 19;10(8):e0135908. doi: 10.1371/journal.pone.0135908 (PMC4546111; doi:10.1371/journal.pone.0135908)
Supplement: S1 Protocol — (PDF) [file pone.0135908.s002.pdf]

Effect of increased convective clearance by on-line hemodiafiltration on all cause and cardiovascular mortality in chronic hemodialysis patients

*THE DUTCH **CON**VECTIVE **TRA**NSPORT **ST**UDY (**CONTRAST**)*

Executive committee:
dr P.J. Blankestijn, internist/nephrologist, UMC (chair)
dr M.L. Bots, epidemiologist, UMC
dr M.A. van den Dorpel, internist/nephrologist, MCRZ-Clara
dr M.P.C Grooteman, internist/nephrologist, VUmc
Prof. dr M.J. Nubé, internist/nephrologist, Medical Center Alkmaar / VUmc
Prof. dr P.M. ter Wee, internist/nephrologist, VUmc (chair)

Departments of Nephrology
University Medical Center Utrecht[1] and Vrije Universiteit Medical Center[2],
[1]Heidelberglaan 100, 3584 CX Utrecht; [2]De Boelelaan 1117, PO Box 7057, 1007 MB Amsterdam; The Netherlands

Correspondence
dr M.P.C Grooteman, internist/nephrologist, VUmc
Department of Nephrology, PO Box 7057, 1007 MB Amsterdam.
The Netherlands

version 1.1
October, 2003

1. CONTENTS 2

2. SUMMARY 4

3. AIM OF THE STUDY 4

4. FLOW CHART 5

5. BACKGROUND 6
- 5.1 cardiovascular disease in hemodialysis patients: introduction
- 5.2 pathogenesis of cardiovascular disease in HD patients
  - 5.2.1 contributing factors
  - 5.2.2 accumulation of ‘uremic toxins’
    - 5.2.2.1 disturbances in the balance between oxidants and anti-oxidants
    - 5.2.2.2 disturbances in the immuno-inflammatory system 7
  - 5.2.3 endothelial dysfunction
  - 5.2.4 evidence/markers of atherosclerosis
- 5.3 technical aspects of hemodialysis and hemodiafiltration 8
  - 5.3.1 hemodialysis
  - 5.3.2 flux, diffusion and convection
  - 5.3.3 hemodiafiltration
- 5.4 potential advantages of techniques with high convective transport
  - 5.4.1 uremic toxins
  - 5.4.2 markers of atherosclerosis 9
  - 5.4.3 cardiovascular stability
  - 5.4.4 cardiovascular and all-cause morbidity and mortality

6. STUDY OBJECTIVES 10
- 6.1 primary objectives
- 6.2 secondary objectives

7. DESIGN OF THE STUDY 11
- 7.1 patients
  - 7.1.1 inclusion and exclusion criteria
  - 7.1.2 participating centers
- 7.2 study approach
  - 7.2.1 stabilisation period
  - 7.2.2 randomisation
- 7.3 end points
  - 7.3.1 primary end points
  - 7.3.2 secondary end points
- 7.4 practical implementation 12
  - 7.4.1 stabilisation period
    - 7.4.1.1 hemodialysis
    - 7.4.1.2 dialysate
    - 7.4.1.3 metabolic control
    - 7.4.1.4 medication
    - 7.4.1.5 randomisation
  - 7.4.2 study period
    - 7.4.2.1 hemodialysis
    - 7.4.2.2 hemodiafiltration
    - 7.4.2.3 metabolic control
    - 7.4.2.4 medication

7.4.3 dialysis procedure
7.4.3.1 dialysate
7.4.3.2 infusate/ the on-line system
7.5 data collection 13
7.5.1 baseline data
7.5.2 recording outcome events
7.5.3 observations during dialysis/hemodiafiltration
7.5.4 left ventricular hypertrophy 14
7.5.5 vessel wall measurements
7.5.6 nutritional state
7.5.7 quality of life
7.5.8 laboratory assessments
7.6 statistical methods 15
7.6.1 primary outcomes
7.6.2 secondary outcomes
7.6.3 sample size considerations
7.6.3.1 primary outcomes
7.6.3.2 secondary outcomes 16
7.6.4 interim analysis
7.7 publication of study results

8. SPECIAL CONSIDERATIONS / ETHICAL ASPECTS 17
8.1 special considerations for the participating patients
8.1.1 possible inconveniences for the patient
8.1.2 possible advantages for the patient
8.1.3 safety measures
8.1.4 research and professional care
8.1.5 participation and informed consent
8.1.6 medical information and data
8.2 special considerations concerning the relevance of the study
8.2.1 scientific relevance
8.2.2 general relevance 18

9. INSURANCE 18

10. TRIAL ORGANISATION 18
10.1 trial coordinating center
10.2 executive committee
10.3 steering committee
10.4 event committee 19
10.5 data monitoring board
10.6 advisory committee

11. LIST OF ADDENDA 20

12. LIST OF ABBREVIATIONS 21

13. REFERENCES 22

2. SUMMARY

Today, an increasing number of patients with chronic renal failure (CRF) is treated with (on-line) hemodiafiltration (HDF). This practice is based on the assumption that the high incidence of cardiovascular (CV) disease, as observed in patients with CRF, is at least partially related to the retention of uremic toxins in the middle and large-middle molecular (MM) range. As HDF lowers these molecules more effectively than HD, it has been suggested that this treatment improves CV outcome, if compared to standard HD.
Thus far, no definite data on the effects of HDF on CV parameters and/or clinical end-points are available. Promising data include a reduction of left ventricular mass index (LVMi) after one year of treatment with acetate free bio-filtration (AFB). Furthermore, relatively high survival rates were reported in a single center non-experimental study on patients who were treated with HDF, if compared to the EDTA registry data on HD-treated patients. Yet, these data are of observational nature, with the possibility of being biased by confounding by indication.
As the accumulation of MMW substances has been implicated in increased oxidative stress and endothelial dysfunction, a reduction of these compounds might improve these derangements. In addition, cardiac dysfunction, atherosclerosis (as measured by left ventricular mass index [LVMi], carotid intima media thickness [CIMT]) and vascular stiffness (as measured by pulse wave velocity [PWV]) might be reduced during HDF, as compared to low-flux HD.
Therefore, we propose a prospective, randomized multicenter trial, comparing (on-line) HDF with HD. After a stabilization period, an expected number of 772 chronic HD patients will be randomized to either HDF or low-flux HD for three years. Primary end points are all cause mortality and combined CV events and mortality. In addition, LVMi, PWV, CIMT and various parameters of oxidative stress, acute phase reaction (APR) and endothelial function will be assessed and compared between treatment groups.
This study will provide strong evidence on the efficacy of HDF compared to low flux HD on CV morbidity and mortality, which is currently lacking but urgently needed. It is highly likely that the outcome of this study will affect current clinical practice considerably, in the Netherlands as well as internationally. Moreover, the study will point towards the mechanisms underlying the effects of HDF.

3. AIM OF THE STUDY

The following hypothesis will be tested:

1. all-cause mortality and combined CV morbidity and mortality in patients treated with (on-line) HDF is lower than in patients treated with standard low-flux HD.
2. a reduction in MMW uremic toxins by HDF leads to an improvement of the ‘uremic profile’ (as measured by AGE-levels, homocysteine levels, oxidative stress, and endothelial dysfunction), if compared to standard low-flux HD.
3. the improvement of the ‘uremic profile’ in HDF-treated patients results in an improvement of endothelial function with a reduction in the progression of vascular injury (as measured by CIMT and PWV) and a reduction in LVMi, if compared to standard low-flux HD.

4. FLOW CHART

Assessment								
Months	0	3	6	12	18	24	30	36
Clinical events	contin	uously						
Routine DGN laboratory assessments	x	x	x	x	x	x	x	x
Study laboratory assessments	x		x	x	x	x		x
LVMi	x		x	x		x		x
CIMT	x			x		x		x
PWV	x		x	x		x		x
Quality of life	x			x		x		x
Nutritional state	x			x		x		x
Medication	x		x	x	x	x	x	x

5. BACKGROUND

5.1 Cardiovascular disease in hemodialysis patients: introduction

Cardiovascular disease (CVD) is the most frequent complication and major cause of mortality in dialysis patients,[1] accounting for more than half of all deaths (70% versus 37% in the normal population). In addition, chronic hemodialysis (HD) patients suffer from atherosclerotic complications at a relatively younger age[2] and die younger from ischemic heart disease.[3] The increased cardiovascular (CV) risk is probably multifactorial in origin and already observed in the pre-dialysis phase.
On theoretical grounds, the pathogenetic process leading to CVD can be divided in 4 stages: 1) contributing factors or risk factors; 2) accumulation of 'uremic toxins' and disturbances in the balance between oxidants and anti-oxidants and the immuno-inflammatory system; 3) endothelial dysfunction; 4) evidence of atherosclerosis.

5.2 Pathogenesis of cardiovascular disease in HD patients

<u>5.2.1 contributing factors</u>
Several conventional risk factors are known to contribute to the increased prevalence and incidence of CVD in chronic HD patients, some being -at least partly- modifiable (smoking, hypertension, dyslipidemia, diabetes), and others not (age, gender). Frequently, a combination of these factors is present, that may explain the high prevalence of CVD in this patient group.[4] Moreover, an impaired renal function per se appears to be a risk factor as well.[5] In this respect, the accumulation of several uremic substances, such as homocysteine and Lp(a), may play an important role. In addition, it has been suggested that HD treatment itself contributes to the micro-inflammatory state that is commonly observed in chronic HD patients (see below).[3]

<u>5.2.2 accumulation of 'uremic toxins'</u>
Apart from urea and creatinin, a variety of 'uremic toxins' has been described in CRF.[6]
Nitric oxide (NO) inhibits key processes in atherosclerosis, such as monocyte adhesion, platelet aggregation and smooth muscle cell proliferation. It has been shown that chronic HD patients exhibit reduced NO levels, as well as an accumulation of *asymmetric dimethylarginine (ADMA),* an endogenous inhibitor of NO-synthase. [7] In fact, ADMA appeared to be a strong predictor of CV events and total mortality in this patient group.[8]
Apart from a characteristic atherogenic *lipid profile*, including low concentrations of high-density lipoprotein (HDL)-cholesterol, chronic HD patients exhibit elevated triglyceride, intermediate density lipoprotein (IDL)-cholesterol and Lp(a) concentrations.[9] In observational studies it was shown that in particular the latter abnormality was related to the presence of CVD in these patients. [10]
Homocysteine (Hcy) is an amino-acid that is produced by the demethylation of methionine. Hcy levels are increased in HD patients, whereas Hcy appeared to be an independent risk factor for CVD in both renal and non-renal patients.[11 12 13] Thus far, it proved difficult to show clinically relevant effects of Hcy lowering therapy. Recently however, it was shown that reduction of Hcy decreased the incidence of restenosis after coronary angioplasty in a non-renal population.[14]
Finally, various other uremic accumulates with potential toxicity have been described[16] , whereas the existence of many *undefined uremic toxins* has been suggested as well. The clinical significance of these substances, however, is unknown.

5.2.2.1 disturbances in the balance between oxidants and anti-oxidants
Oxidative stress can be defined as an imbalance between antioxidant and oxidant generating systems. There is accumulating evidence that CRF is associated with enhanced oxidative stress.[15 16] It has been shown that the blood-membrane interaction during HD triggers circulating neutrophils to produce reactive oxygen species (ROS).[17 18] Both the microbiological quality of the dialysate, the presence of co-morbidity and specific medication might be involved as well. Accumulating evidence has indicated that oxidative stress plays

an important role in the pathogenesis of atherosclerosis.[19 20] In CRF, endothelial dysfunction appeared to be related to the degree of oxidative stress.[21]
Oxidative stress also promotes the generation of advanced glycation end products (AGEs), which are formed by non-enzymatic processes between reactive amino-groups of proteins, peptides or amino-acids and a ketone or aldehyde group of reducing sugars (*carbonyl stress*). AGEs accumulate with advancing age, diabetes mellitus and renal failure.[22 23]
Recent evidence indicates that the accumulation of these substances might play an important role in the development of accelerated atherosclerosis in CRF patients with and without diabetes mellitus (DM).[24 25] Furthermore, it has been suggested that AGEs contribute to the micro-inflammatory process in this patient group.[26]

5.2.2.2 disturbances in the immuno-inflammatory system
Numerous observations have led to the 'response to injury' hypothesis of atherosclerosis.[27] From the earliest lesion until the occurrence of CV, an inflammatory component appears to be involved in its pathogenesis. The prototypical marker of inflammation, or the acute phase response (APR) is C-reactive protein (CRP). CRP values are correlated with CV morbidity and mortality in both the normal population[28] and chronic HD patients.[29 30] In the latter group, elevated CRP levels are frequently observed,[31] and might be involved in the process of accelerated atherosclerosis.[32] In both renal and non-renal patients, an elevated CRP was related to the extent of carotid atherosclerosis (carotid intima media thickness CIMT),[33 34] and to the absence of an improvement in arterial stiffness (pulse wave velocity PWV) in response to blood pressure (BP) treatment.[35] The cause of the apparent micro-inflammatory state in chronic HD patients is not exactly known. Clinical studies suggest that the dialyser membrane material[36] and/or clinical events[37] play a significant role, while laboratory studies emphasise the importance of monocyte activation due to backtransport of contaminated dialysate. Since an elevation of pro-inflammatory cytokines and/or CRP is already observed early in CRF,[38 39] factors related to uraemia per se appear to be involved as well.

5.2.3 endothelial dysfunction
Endothelial dysfunction is the dysregulation of homeostatic mechanisms, which normally operate in healthy endothelial cells. Dysfunction of the endothelium is a critical factor in the pathogenesis of vascular disease.[40] A variety of endothelial cell derived products, including circulating adhesion molecules, are candidates for monitoring the condition of the endothelium.[41] High serum levels of adhesion molecules may predict future CVD.[42]
Whether induced by classical bio-incompatibility, monocyte activation and/or oxidative and carbonyl stress, substantial evidence indicates that the HD procedure itself induces damage of the endothelium. A number of studies showed an increase in *thrombomodulin,*[43] *vWF,*[44] *sICAM-1, sVCAM-1*[45] *and E-selectine* during clinical HD.[46] Furthermore, markers of endothelial (dys)function seem to be related to both the APR [42] and carotid IMT (CIMT).[47 48]

5.2.4 evidence / markers of atherosclerosis
At the initiation of renal replacement therapy, 74% of the patients show *left ventricular hypertrophy (LVH)* on echocardiography.[49] LVH is an independent CV risk factor both in renal[50 51] and in non-renal patients.[52] Evidence has been obtained that stabilization or regression of LVH improves the prognosis.[53 54] LVH in chronic HD patients is influenced by many factors, such as high BP,[55] anaemia[56] and parathyroid hormone levels.[57]
The extent of atherosclerosis, as found in the common carotid artery, shows a consistent and significant relation with coronary artery disease.[58] The *CIMT* is an independent risk factor for CV events in the non-renal population[59 60] and a predictor of CV death in dialysis patients.[61] In these patients, CIMT is increased as compared to normal controls.[62 63] In cholesterol and BP lowering trials in non-renal patients, regression of CIMT has been described.[64 65]
Aortic *PWV* is also a marker of individual CV risk,[66] reflecting arterial stiffness. PWV proved to be an independent predictor of CV mortality in patients with high BP[67] and dialysis patients.[68 69] In addition, it appeared to be related to the amount of vascular calcifications.[70] The prognostic value of aortic PWV measurements in patients with CRF was further

demonstrated in a cohort of 150 patients. [35] Subjects who received an ACE-inhibitor for BP lowering and whose aortic stiffness improved had a considerably higher survival rate compared to those subjects whose BP was lowered, but whose aortic stiffness remained unaltered.

5.3 Technical aspects of hemodialysis and hemodiafiltration

5.3.1 hemodialysis

Today, HD is the most common used renal replacement therapy worldwide. The main objective is the removal of excess fluid and toxic solutes from the patient.[71] Despite the relative efficiency of modern dialysers, HD remains inferior to normal kidney function. [72] Not only small molecules, such as creatinin and urea, but also so-called ‘middle molecules’ as well as some larger substances are cleared only inadequately by HD. Hence, ‘uremic toxins’ accumulate in chronic HD patients. In addition, undesirable interactions occur between the patient and the various components of the extra-corporeal-circuit (ECC), termed ‘bio-incompatibility’ (BI).[73]

5.3.2 flux, diffusion and convection

In the Netherlands, standard HD treatment consists of bicarbonate dialysis with low- or high-flux dialysers. Small MW substances (< 500 D) are cleared almost exclusively by diffusion, driven by the concentration gradient between blood and dialysate. The most important clearance route for middle and large-middle MW (2-50 kD) substances is by convection, occurring passively with the flux of water through the membrane. During low-flux HD (ultrafiltration [UF] coefficient < 10 ml/mmHg/h), solutes are almost exclusively cleared by diffusion. Convective transport is practically zero, since UF is restricted to the required weight loss and the membrane prohibits the sieving of larger solutes. Apart from small MW substances, small middle MW (MMW) substances (500–2000 D) are cleared by diffusion as well.[74] In high-flux HD (UF coefficient > 20 ml/mmHg/h), solutes are cleared by both diffusion and convection. Total UF can surpass the required weight loss by internal filtration, which increases convective transport to a variable amount (ca 9 l/session).[75 76] Finally, clearance of MMW molecules occurs to a variable extent by adsorption onto the dialyse membrane.[77]

5.3.3 hemodiafiltration

During hemodiafiltration (HDF), fluid removal exceeds the desired weight loss of the patient. Fluid balance is maintained by the infusion of a pyrogen-free solution. Dialysate is used to create a concentration gradient for solute removal by diffusion, as in standard HD. The clearance of larger solutes is increased by using excess UF to provide solute removal by convection. [78] In recent years, on-line preparation of infusate has become available for clinical practice.[79 80] With this modality, the volume of substitution fluid and hence UF rate can be increased considerably (up to 60 l per treatment), without increasing costs or handling of prepared fluid bags. Thus, the amount of MMW molecules removal occurs as follows: low flux HD < high flux HD < HDF.

5.4 Potential advantages of techniques with high convective transport

5.4.1 uremic toxins

Age-peptides: High-flux membranes appear to be more effective in the removal of AGE-peptides than conventional low-flux dialysers (reviewed in [81]). Interestingly, in a long term study with super-flux dialysers (UF coefficient > 60 ml/mmHg/h), serum AGE levels were lowered to the largest extent, if compared to conventional low flux and high flux dialysers.[82 83] These results suggest a significant influence of convective transport on AGE-levels, which might be mediated by the removal of uremic toxins promoting AGE-formation. In contrast, a cross-sectional study on high-flux HD and HDF showed comparable AGE-levels. [84]
Lipids: After HD with high flux dialysers the atherogenic profile of chronic HD patients improved [18 19] , due to a decline in triglyceride levels[85] and Lp(a)[86] values, whereas these levels were unchanged after HD with low flux HD.[87 88 89] Because triglycerides can not be cleared by the dialyser, another mechanism, such as the removal of a dialyzable factor

influencing triglyceride metabolism,[82] possibly low-molecular weight AGEs,[90] might explain these results.
Homocysteine: Although Hcy levels were reduced after a single HD treatment,[91] pre-dialysis values remained stable after 3 months of high-flux HD.[92] In contrast, during HD with super-flux membranes, Hcy levels were reduced in the long term,[93] suggesting a role for convective clearance of uremic toxins influencing Hcy-metabolism. The effect of HDF on Hcy-levels is not known.
CRP: The effects of dialyser membrane material[94] and/or clinical events[37] on CRP levels in chronic HD patients seem to outweigh the effects of flux characteristics in clinical HD. In HDF higher UF volumes seem to be related to less cytokine production[95] and/or lower CRP levels, if compared to lower UF-volumes.[96] Whether this phenomenon is related to the removal of undefined uremic toxins and hence convective transport remains to be established.

5.4.2 markers of atherosclerosis

As far as we know, the effect of different dialysis techniques on CIMT and aortic PWV has not been investigated so far. In a recent, small prospective randomised study, a 17 % decrease in left ventricular mass index (LVMi) was shown during acetate free biofiltration (AFB), in contrast to an 18% increase in LVMi after 1 year of high flux HD. [97]

5.4.3 cardiovascular stability

Symptomatic hypotension is the most frequent intra-dialytic complication during clinical HD. Several studies suggest that dialysis techniques, which are based on convective transport, provide better hemodynamic stability than diffusive techniques.[98] [99] This effect seems not to be mediated by differences in dialyser material, type of dialysate, sodium balance or rate of solute removal.[100] Furthermore, since HDF and hemofiltration (HF) appear to offer comparable hemodynamic stability, neither the presence nor the absence of dialysate seems to play a role. The difference in heat balance and subsequent vasoconstriction during dialysis with convective techniques might account for the better hemodynamic stability.[101] [102] [103]

5.4.4 cardiovascular and all-cause morbidity and mortality

Large retrospective studies have clearly shown that HD with high flux dialysers results in both lower morbidity and mortality than HD with low flux devices.[104] [105] [106] However, in most of these investigations biocompatible high-flux membranes were compared with low-flux bio-incompatible materials. Hence, it remains unclear whether the flux or biocompatibility characteristics of the dialyser explain these differences.
In two recent observational studies, a beneficial effect of convective clearance on patient survival was suggested. [107,108] However, although the latter studies encompass large numbers of patients, the survival benefit was not significant or very small. Moreover, information on dialyser biocompatibility was not available. Recently, relatively high survival rates were reported in patients who were treated with on-line HDF, if compared to the EDTA registry data.[109] However, both retrospective and prospective observational studies are subject to bias through a confounding by (contra) indication mechanism: i.e., the assignment to HDF is related to having either a high risk (indication) or having a low risk of future events (contra-indication). As a consequence, HDF patients may not have a similar risk profile at the start of the study, if compared to non-HDF patients. Finally, a small prospective randomised study (n=44) , comparing low flux HD with on-line HDF failed to show any effect of treatment on clinical parameters and survival.[72] Yet, this study was too small to provide definite conclusions on the efficacy of HDF. Lastly, in a prospective trial (HDF: n=50; high-flux HD: n=51;low-flux HD: n=279), no differences in treatment tolerance, nutritional status and patient survival were observed.[110] However, the latter results are debatable as the number of patients in each group was relatively small and the follow-up period rather short. Taken together, so far no reliable data are available on the effect of HDF on survival and morbidity in patients with CRF.

Therefore, based on the above-mentioned theoretical considerations, the outcome of surrogate end-points and the lack of reliable data on morbidity and mortality, in combination with the growing interest in convective techniques under nephrologists, a randomized prospective study of sufficient sample size is proposed.

6. STUDY OBJECTIVES

6.1 Primary objectives:

- To assess the effect of on-line HDF on fatal and non-fatal cardiovascular events, if compared to standard low-flux HD.
- To assess the effect of on-line HDF on all cause mortality, if compared to standard low-flux HD.

6.2 Secondary objectives:

- to assess the effect of on-line HDF on the progression of left ventricular hypertrophy, as assessed by echocardiography, if compared to standard low-flux HD;
- to assess the effect of on-line HDF on the progression of atherosclerosis as assessed by measurement of carotid intima-media thickness, if compared to standard low-flux HD;
- to assess the effect of on-line HDF on the progression of arterial stiffness, as assessed by measurement of aortic pulse wave velocity, if compared to standard low-flux HD;
- to assess the effect of on-line HDF on endothelial function, as assessed by vWF, sICAM-1, sVCAM-1 and s-E selectine , if compared to standard low-flux HD
- to assess the effect of on-line HDF on the micro-inflammatory state, as assessed by CRP, if compared to standard low-flux HD;
- to assess the effect of on-line HDF on lipid profiles, as assessed by cholesterol, HDL-cholesterol, triglycerides and Lp(a) , if compared to standard low-flux HD;
- to assess the effect of on-line HDF on markers of oxidative stress, as assessed by oxidized LDL, 2-OH deoxyguanosine, asymmetric dimethyl arginine (ADMA), 8-isoprostane, carboxymethyllysine (CML), TBARS and GSSG/GSH, if compared with low-flux HD;
- to assess the effect of on-line HDF on various other uremic toxins, as assessed by homocysteine and ß-$_2$-microglobulin , if compared to standard low-flux HD;
- to assess the effect of on-line HDF on quality of life, as assessed by a questionnaire, if compared to low-flux HD;
- to assess the effect of on-line HDF on nutritional state, as assessed by pre-albumin, dry weight and subjective global assessment (SGA), if compared to low-flux HD.

7. DESIGN OF THE STUDY

The study is designed as a open, parallel group, randomised controlled intervention study.

7.1 Patients

7.1.1 Inclusion and exclusion criteria

Inclusion criteria are:
- patients treated by HD 2 or 3 times a week, for at least 2 months.
- patients able to understand the study procedures.
- patients willing to provide written informed consent.

Exclusion criteria are:
- current age < 18 years*
- treatment by HDF or high flux HD in the preceding 6 months
- severe incompliance**
- life expectancy < 3 months due to non renal disease
- participation to other clinical intervention trials evaluating cardiovascular outcome***

* In Europe, the median age at the start of HD therapy was 60 years (proportion of new HD-patients 60-69 yr: 25%, >70 yr: 27%).[111] Thus, *chronic* HD patients tend to be still older. Since the study results might be of importance for HD patients of all ages, it seems irrational to exclude patients > 70 years. Thus, no upper age limit is applied.
** severe non-adherence to the dialysis procedure and accompanying prescriptions, especially frequency and duration of dialysis treatment and fluid restriction.
*** Participation of patients to other (e.g. observational) studies will be discussed with and decided by the executive committee.

7.1.2 Participating centers

All dialysis centres which are able to perform on-line hemodiafiltration in the Netherlands will be asked to participate in the study to obtain a total patient number of 772. As of yet, 17 centres have agreed to participate in the study. Participating centres should include a minimum of 10 patients.

7.2 Study approach

7.2.1 stabilisation period

Before randomisation, patients will be dialysed on low-flux synthetic dialysers. During this period, treatment times will be determined in order to achieve an adequate dialysis dose, according to DGN-standards (target dialysis spKt/V ≥ 1.2/treatment).

7.2.2 randomisation

As soon as patients are considered to be ‘stable’, patients will be randomized by the Julius Center Randomisation office. Patients will be randomized into a 1:1 ratio for treatment with on-line hemodiafiltration (HDF) or continuation of treatment with low-flux hemodialysis (HD) during 3 years. Randomisation will take place stratified by participating clinic.

7.3 End points

7.3.1 Primary end points
- non-fatal and fatal cardiovascular events
- all cause mortality

7.3.2 Secondary end points

Changes in:
- carotid intima media thickness (cIMT)
- aortic pulse wave velocity (PWV)
- left ventricular mass index (LVMi)

- interdialytic blood pressure
- laboratory assessments (routine – according to DGN guidelines; oxidative stress; acute phase response; lipid profile; various)
- quality of life (QoL)
- nutritional state

7.4 Practical implementation

7.4.1 Stabilisation period

7.4.1.1 Hemodialysis: patients will be dialyzed 3 times (or 2 times) a week with a synthetic low-flux membrane (UF-coefficient < 12 ml/mmHg/h, sterilization by steam or γ-irradiation) during the stabilisation phase. Blood flow will be maintained at 250-300 ml/min. Anticoagulation is performed with low molecular weight heparin (LMWH) before HD. Patients on coumarins will receive 50% of the LMWH dose. Treatment times will be adapted to a target dialysis spKt/V urea of ≥1.2 / treatment.

7.4.1.2 Dialysate: Ultrapure water is used for preparation of dialysis fluid. Bicarbonate is provided from powder cartridges to avoid the risk of a bacterial load from bicarbonate concentrates. For instance, the biBAG[R] system (Fresenius) and BiCart[R] system (Gambro) will be used. The dialysate flow is 500 ml/min in HD. The temperature of the dialysate is 36°C.

7.4.1.3 Metabolic control: metabolic control will be performed according to the guidelines of the Nederlandse Federatie voor Nefrologie / Kommissie Kwaliteitsbewaking (NFvN/KK: anemia / calcium- and phosphate levels / acidosis).
7.4.1.4 Medication: antihypertensive medication, lipid lowering therapy, inhibition platelet aggregation and medication to treat renal anemia and renal osteodystrophy will be prescribed according to the NFvN/KK guidelines, and if not available according to common practice.

7.4.1.5 Randomisation: After the stabilisation period, patients will be randomised centrally to either treatment with low-flux HD, or treatment with on-line HDF (1:1). Stratification will be performed for the participating centres.

7.4.2 Study period
7.4.2.1 Hemodialysis: HD treatment, as performed in the stabilisation period, will be continued. Treatment times will be adjusted only of dialysis spKt/V urea < 1.2 / treatment.

7.4.2.2 Hemodiafiltration: Patients will be treated with (on-line) HDF, target dose postdilution 6 l/h (~100 ml/min) and a high-flux synthetic dialyser (UF-coefficient > 20 ml/mmHg/h, sterilization by steam or γ-irradiation). Blood flow, anticoagulation and treatment times will be fixed according to the prescription in the stabilisation period. If the blood flow is less than 300 ml/min, the postdilution volume will be decreased accordingly (filtration and postdilution <33% of blood flow). If necessary, LMWH will be given in two separate doses. The treatment time will be adjusted only when spKt/V urea is < 1.2 / treatment.
7.4.2.3 Metabolic control: see ‘stabilisation period’.
7.4.2.4 Medication: see ‘stabilisation period’.

7.4.3 Dialysis procedure:
7.4.3.1 Dialysate: ultrapure water is used for preparation of bicarbonate-containing dialysis fluid, which undergoes one step of ultrafiltration converting it into ultrapure dialysis fluid. Dialysis fluid is produced at a rate of 600-800 ml/min of which approximately 100 ml/min is diverted for further processing into substitution fluid. The electrolyte composition of the dialysis fluid is: Na 138-140 mmol/l; K 1.0-3.0 mmol/l; HCO_3 30-35 mmol/l; Ca 1.0-1.7 mmol/l; Mg 0.5 mmol/l; Cl 108-109.5 mmol/l; glucose 0-5.6 mmol/l; acetate 3 mmol/l.
7.4.3.2 Infusate/ the on-line system: The substitution fluid is prepared from the dialysis fluid by one additional step of controlled ultrafiltration, before it is infused post-filter into the blood. The electrolyte composition of the substitution fluid is the same as the composition of the

dialysis fluid. Ultrafiltration procedures will be performed according to the manufacturers' instructions. For instance:
- the on-line system, ONLINEplus™ (Fresenius Medical Care, Bad Homburg, Germany) is integrated into the dialysis machine (4008 series; Fresenius Medical Care) and consists of two ultrafilters (DIASAFE® plus), an infusate pump module, and disposable infusate lines. Infusate is prepared continuously by double-stage ultrafiltration. Both filters are subjected to automated membrane integrity tests before dialysis, and are replaced after 3 months of use. Dialysis fluid downstream from the first filter stage enters the dialyser; part of the stream is subjected to cross-flow filtration in the second filter in order to produce infusate. The infusate stream is connected with the venous bubble catcher for post-dilutional HDF.
- an AK 100/200 ULTRA dialysis machine (Gambro AB, Lund, Sweden) prepares ultrapure water and ultrapure dialysis fluid by stepwise ultrafiltration of water and bicarbonate – containing dialysis fluid (BiCart) using two polyamide ultrafilters (U 8000 S). When used for HDF, sterile non-pyrogenic solution is prepared on-line from the ultrapure dialysis fluid by an additional step of ultrafiltration using a sterile polyamide ultrafilter (U2000) integrated in a sterile line set (Steriset). The hygiene of the fluid pathway, including the U8000S ultrafilters, will be assured by heat disinfection after each treatment. The U8000S filters are changed bimonthly. The final ultrafilter (U2000) is employed on a single-use basis.

7.5 Data collection

7.5.1 Baseline data

Medical information will be obtained from the medical records and nephrologists in charge before the start of the study: i.e. demographical data, cardiovascular risk factor information, time on dialysis, cause of renal insufficiency, medical history (co-morbidity), and medication. A dedicated case report form to asses baseline data will be provided. This form is designed in teleform, which allows to put the information on the sheet directly into a study database (Teleform). Thus, in principle no data entry by typing is needed.

7.5.2 Recording outcome events

Follow-up will be performed through the nephrologist, since the patients are seen at their clinic 2 or 3 times a week. Every 3 months, the investigators will visit the participating centers, where they will interview the nephrologists personally and review the case records of the patients on the occurrence of CV events or death.

CV events include a fatal or non-fatal myocardial infarction, stroke, lower extremity arterial disease, therapeutic coronary procedure (PTCA/stenting), therapeutic carotid procedure (endarteriectomy/stenting), vascular surgery (revascularisations), and angina pectoris. Congestive heart failure is excluded, since the discrimination with fluid overload is often hard to make.

Furthermore, hospitalisations, the duration of the hospitalisations and main diagnosis (including the occurrence of infections) will be recorded during the study period.
When an event has occurred, the date of the event is being recorded and information on the event is copied from the medical records for review by an event committee. Events will be coded independently from information on the received treatment. Events will be coded as fatal and non-fatal, definite, probable and possible and not codeable (i.e., insufficient information). Only definite and probable events will be used for the analysis. This procedure is currently successfully applied in a number of studies coordinated by the Julius Center, e.g. in the SMART study.[112] Data forms from the SMART study will be used and changed for the current trial if needed.

7.5.3 Observations during HD/HDF

Dry weight and UF volume will be recorded for each treatment, as well as the achieved filtration/substitution dose per treatment.

7.5.4 Left ventricular hypertrophy

Left ventricular mass index (LVMi) will be assessed by echocardiography at baseline, and after 6 months, 12 months and annually afterwards, on a midweek non-dialysis day. Left ventricular end diastolic diameter (LVEDD), left ventricular ejection fraction (LVEF), posterior wall and septal thickness will be determined as well, and recorded on videotape. Videotapes will be evaluated centrally by experienced cardiologists, blinded for the treatment modality.

7.5.5 Vessel wall measurements

With respect to carotid intima-media thickness (CIMT), the outcome is the change in mean common CIMT, defined as the average of the intima-media thickness measurements performed circumferentially at pre-defined angles for the near and far wall of 10 mm segments of the right and left distal common carotid arteries, the far wall of the bifurcation and the far wall of the internal carotid artery. A limited number of centers is going to be involved in the CIMT measurements in this study. Centers will be trained according to a central uniform carotid ultrasound protocol. Before actually starting the study, sonographers need to be certified as outlined in the CIMT ultrasound protocol. Measurements will be performed at baseline and then annually on a midweek non-dialysis day.The ultrasound scan is being recorded on videotape and analyzed off line by a core laboratory. QA/QC procedures as existing and applied in several (inter)national trials will implemented.

Pulse wave velocity (PWV) is determined to provide additional information on functional changes of the arterial wall. The outcome measurement is the change in aortic PWV. A limited number of centers is involved in the PWV measurements in this study. Centers will be trained according to a central uniform PWV protocol. Before actually starting the study, technicians need to be certified as outlined in the protocol. Measurement data are checked regularly on quality control aspects as defined in the protocol (see appendix). Measurements will be performed at baseline and then annually on a midweek non-dialysis day.

7.5.6 Nutritional state

At base-line, after 1, 2 years and at the end of the study, nutritional state is assessed by subjective global assessment (SGA),[113] pre-albumin and dry weight.

7.5.7 Quality of life

Patient well-being will be estimated at base-line, and once a year by the Kidney Disease Quality of Life Short Form (KDQOL-SF), which is validated for dialysis patients.[114 115]

7.5.8 Laboratory assessments

At base-line, and after 6, 12, 18 months and then annually, blood samples will be drawn from the arterial line before a dialysis session. Patients will be fasting, and the samples will be drawn before the administration of low molecular weight heparin (LMWH). In these samples, the following markers will be assessed:

- routine assessments, as indicated by the DGN: Hb, Ht, phosphate, calcium, parathyroid hormone (PTH); spKt/V urea (monthly), residual renal function (expressed as the mean of urea and creatinin clearance in an interdialytic 24 hour urine collection; every 3 months).
- acute phase response: C-reactive protein (CRP)
- lipid profile: low density lipoprotein (LDL)-cholesterol, high-density lipoprotein (HDL)-cholesterol, Lp(a), triglycerides
- oxidative and carbonyl stress: circulating oxidized low-density lipoprotein (oxLDL), carboxymethyllysine (CML), asymmetric dimethylarginine (ADMA), 2-OH deoxy guanosine, 8-isoprosatane, GSSH/GSH, TBARS

- endothelial dysfunction: von Willebrand factor (vWF), soluble intercellular adhesion molecule (sICAM-1), soluble vascular adhesion molecule (sVCAM-1)
- various: total homocysteine (Hcy), $ß_2$-microglobulin ($ß_2$-m), pre-albumin

A detailed protocol concerning the collection, preparation, processing , short-term and long-term storage as well as archiving is currently being developed. A present citrate, EDTA and serum sample will be stored for later determinations. In addition, buffy-coat will be stored for future research possibilities on the genetic effects on the response to HDF, after permission of the patients (in the informed consent form).

7.6 Statistical methods

7.6.1 Primary outcomes

The results of the study will be analysed following the ‘intention to treat’ principle. This means that for the analyses subjects will remain in the group they have been allocated to by the randomisation. Results will be presented as Kaplan-Meier curves for the two treatments and the difference between the treatments will be analysed using a log-rank test. For the primary outcome variables the log-rank test will be adjusted for the effect of the cumulative data analyses. Results will be presented for all cause mortality and CV events separately.
An exploratory analysis will be performed using the ‘on treatment’ approach, i.e., analyses in which subjects are being categorised to the actual treatment they have received.

7.6.2 Secondary outcomes

The primary analysis of CIMT progression will employ a linear random coefficient (Laird-Ware) model using real visit days, treatment and clinical center as independent variables. For each participant, the intercept and slope of CIMT change over time is assumed to be a normally distributed random variable with different means for the two treatment groups. The mean slope for the HDF treatment group will be compared to that for the low flux group using linear contrasts and a 5% significance level. All analyses will be based on an intention-to-treat approach, although a per-protocol sample will also be examined. Additional exploratory analyses will evaluate the impact of including baseline IMT, lumen diameter, ultrasound reader, and center as additional co-variates.

The data analytic approach to arrive at the PWV outcome variable and the LVM outcome variable is similar to that of the CIMT outcome. Adjustments that will be taken into account in the estimates are changes in MAP and changes in heart rate, since both are closely related to PWV.

7.6.3 Sample size considerations

7.6.3.1 Primary outcome

The sample size of the present study is based on the following event rates: the 3 year all cause mortality rate among subjects with ESRD is 44% based on RENINE data. CV mortality constitutes 40-60% of the total group of deaths, leading to a 3 year CV mortality rate of 22% in HD patients. Assuming that the incidence of non-fatal CVD is equal to the CV mortality rate (22%), we assume a three year incidence of fatal and non-fatal CVD of 44%. In addition, based on experience around 8% of the ESRD patient will undergo renal transplantation yearly and as such is being censored in the trial.
Assuming that HDF will reduce all cause mortality with 20%, we estimate that with a two-sided alpha of 0.05 and a power of 80%, about 772 patients need to be enrolled and followed for three years. In these patients we expect about 250 events to come to a decision.
Assuming that HDF will also reduce the incidence of fatal and non-fatal CVD with 20%, we estimate that with a two-sided alpha of 0.05 and a power of 80%, the same amount of patients need to be enrolled and followed for three years. (Note that the total number of patients to be included can not be specified in advance because of the planned sequential [interim] analyses.)

7.6.3.2 Secondary outcomes
The sample size consideration with respect to measures of vascular damage (atherosclerosis) are based a number of issues:

- The estimated progression rate in HD patients
- The estimated standard deviation of the individual progression rates
- The effect the treatment would have on the progression rates.

For CIMT and PWV these estimates are not available for HD patients, so we have to rely on data from other patients, e.g. patients with coronary heart disease.

For common CIMT among subjects with hyperlipidemia or hypertension or subjects with previous CV disease a median annual progression rate in CIMT of 0.021 mm/yr was found in the placebo groups of randomised controlled trials. Because CIMT progression rates for HD patients are lacking, data on the standard deviation of the CIMT progression estimate for an individual is also lacking. From the overview of placebo groups of several randomised controlled trials, a median standard deviation for the common CIMT was found of 0.053 mm/yr. With these estimates, we are able to detect a treatment effect of 80%, with a power of 80% and a two-sided alpha of 5% using 155 patients per treatment arm.

The above mentioned data for PWV are even more limited than that for CIMT. Based on cross-sectional data among healthy men aged 40-80 years, an increase in PWV per year is around 0.42 m/s. The standard deviation of repeated PWV measurements, as an estimate of the progression rate, is 1.0 m/s. With these estimates, we are able to detect a treatment effect of 80%, with a power of 80% and a two-sided alpha of 5% using 138 patients per treatment arm.

The above mentioned data for LVMi are even more limited than for PWV and CIMT. Based on two observational studies among patients who were on dialysis, [54,116] the estimated mean change in LVMi, assessed using 2D echocardiography in a 3 year period is around 10 g/m^2 with an estimated SD of 47. With a sample size of 155 subjects per treatment arm as needed for CIMT measurements, we will be able to detect a treatment effect of 150% with a power of 80% and a two-sided alpha of 5%.

<u>7.6.4 Interim analysis</u>
In this study, sequential (interim) analyses will be performed. The reason for this approach is that on average less patients are needed in the study when the expected difference in the primary outcome variable is real or when no difference can be expected anymore. Sequential analyses are performed on survival outcome variables according to the double triangular test as described by Whitehead[117] and implemented in the computer programme PEST version 4.[118] The sequential (interim) analyses will be performed by an independent data monitoring board (DMB).

This DMB will be initiated to evaluate the findings of the interim analyses. The DMB consists of a biostatistician (chairman), a nephrologist, an internist, a vascular surgeon and a clinical epidemiologist. The steering committee will provide the DMB every 2 months with the relevant database to perform the unblinded analyses. The main task of the DMB is to decide whether the analyses provide definite proof of either efficacy or no efficacy with respect to the primary outcome. The limits upon which these decisions are to be decided have been defined at the start of the study by the steering committee, advised by the biostatistician I. van der Tweel.

7.7 Publication of the study results
The results of the study will be published in peer reviewed, international medical (nephrology) journals. Possible sponsors will not have influence on the publication of the results, irrespective of their nature.

Main findings will be written by the executive committee members and published by the executive committee members on behalf of the study group. All other papers will be published by various authors, on behalf of the study group. Papers additional to the main research question will be discussed in the executive committee. Writing groups will be assigned, with one person taking the lead and being mainly responsible.

8 SPECIAL CONSIDERATIONS / ETHICAL ASPECTS

8.1 special considerations for the participating patients

8.1.1 possible inconveniences for the patient: During the study, patients will be dialyzed according to their previous prescription times at their usual treatment days and time. During the study, they will be treated with either HD, or HDF. Neither treatment is an extra burden to the patient. Once a year, the following investigations will be performed, preferentially on the same day: echocardiography, vessel wall measurements, assessment of nutritional state and quality of life.
Blood samples will be taken just before dialysis from the blood lines, in order to avoid extra vena-punctures. The amount of blood taken for analytical purposes might be an inconvenience for the participants of the study. However, since HD patients are kept at a stable hemoglobin (Hb) level of about 7.0 mmol/l with erythropoietin, any possible decrease in Hb will be compensated for.

8.1.2 possible advantages for the patient: Patients might be feeling better by the investigational treatment. Their phosphate levels might be somewhat lower, resulting in less extra-ossal calcifications and itching. Today, generally, nephrologists treat patients with high volume HDF only if they suffer during dialysis from hemodynamic instability, or from itching. However, if the superior clearance of MMW substances by HDF is clinically relevant, treatment with HDF might show important benefits on the long term (dialysis related amyloidosis, atherosclerosis, and other complications of the uremic syndrome).

8.1.3 safety measures: clinical HD is a well-controlled treatment. One specialized dialysis nurse is taking care of two or three patients. Regular checks (1-2/hr) of blood pressure, pulse rate and clinical well being are performed.
For on-line HDF, strict guidelines for dialysis fluid purity are mandatory. The production of ultrapure water should be ascertained by detailed quality standards. The chemical and bacteriological purity of the dialysate will be monitored routinely and regularly. All participating centres will adopt a functioning protocol for quality control, and register their results.

8.1.4 research and professional care: all participants can withdraw freely from the study whenever they want. Withdrawal will not interfere with their medical care. Participation to the study will be stopped if the clinical situation suggests any treatment-related problem. On-line HDF is already applied in several centres in The Netherlands and Europe.

8.1.5 participation and informed consent: Patients treated with HD in the participating centres will be asked to participate in the study by the investigators. Participation is possible after giving written informed consent.

8.1.6 medical information and data: Medical information about the patients and data collected for the study are confidential, and only available to the investigators and doctors involved in the medical care of the patients.

8.2 special considerations concerning the relevance of the study

8.2.1 scientific relevance: In chronic HD patients both signs of an APR, increased oxidative stress and endothelial dysfunction have been described extensively. These phenomena

appear to be related to atherosclerosis, which is one of the main clinical problems in this patient group. Therefore, it is of utmost importance to unravel the mechanisms responsible for these phenomena and to look for dialysis modalities which are able to attenuate these side effects of HD. If on-line HDF provides an improvement in CV morbidity and mortality, this means a breakthrough in the treatment of CRF patients, and in the understanding of uremic toxicity.

8.2.2 general relevance, worldwide more than a half million patients depend on HD for their survival. However, as mentioned before, chronic HD patients are subject to high morbidity and mortality, which seems mainly due to the fact that HD treatment is inferior to normal kidney function. Thus, a continuous effort is made to improve dialysis therapy, in particular by the development of dialysis techniques which provide higher convective clearance. Based on current knowledge, it is to be expected that both survival and quality of life of chronic HD patients will improve substantially in the forthcoming years.

9. INSURANCE

The Medical Ethical Committee of the VU Medical Center decided that the present study poses no risks of any harm or death or injury to the participants. Hence, exemption of insurance was provided. Participants in the study will be informed about this decision by the patient information letter.

10. TRIAL ORGANISATION

10.1 Trial coordinating centre

The trial coordinating center will be located in the Vrije Universiteit Medisch Centrum, Amsterdam, and project management and data mangement will be performed by the Julius Center, Utrecht Medisch Centrum, Utrecht. The data management approach taken in this trial is similar to the one used in the SMART-study,[112] but of course dedicated and modified specifically to the current trial. The trial coordinating center consists of:
dr M.P.C. Grooteman, internist / nephrologist, VUmc (study coordinator)
dr M.L. Bots, epidemiologist, Julius Center, UMC (data manager)
E. Ram, Julius Center, UMC (project manager)

10.2 Executive committee

The nephrologists and epidemiologist who initiated the study, as well as the two research physicians (one vacant) will constitute the executive committee. The executive committee will meet regularly and decide on practical issues concerning the study. Furthermore, this committee will inform the participating nephrologists on the progress of the study. Members of the executive committee are:
dr P.J. Blankestijn, internist/nephrologist, UMC (chair)
dr M.L. Bots, epidemiologist, UMC
dr M.A. van den Dorpel, internist/nephrologist, MCRZ-Clara
dr M.P.C Grooteman, internist/nephrologist, VUmc
prof. dr M.J. Nubé, internist/nephrologist, Medical Center Alkmaar / VUmc
prof. dr P.M. ter Wee, internist/nephrologist, VUmc (chair)
research physicians:
drs E.L. Penne
one research physician vacant

10.3 Steering committee

The steering committee consists of nephrologists of the participating centers. Members of the steering committee are:
dr J.J. Beutler, Jeroen Bosch Medicentrum, ’s Hertogenbosch

dr W.H. Boer, Utrecht Medisch Centrum, Utrecht
dr E.F.H. van Bommel, Albert Schweitzer Ziekenhuis, Dordrecht
mevr. dr M. van Buren, Leyenburg Ziekenhuis, Den Haag
dr. A. van Es, Ziekenhuis Hilversum, Hilversum
dr G.W. Feith, Gelderse Vallei Ziekenhuis, Ede
dr W. Bax, Medisch Centrum Alkmaar, Alkmaar
dr A.B. Geers, Sint Antonius Ziekenhuis, Nieuwegein
J.O.. Groeneveld, Onze Lieve Vrouwe Gasthuis, Amsterdam
W.P. Haanstra, Scheper Ziekenhuis, Emmen
dr H.W. van Hamersvelt, Universitair Medisch Centrum St Radboud, Nijmegen
F. de Heer, Maaslandziekenhuis, Sittard
C.T. op de Hoek, Sint Fransiscus Gasthuis, Rotterdam
dr M.P. Kooistra, Stichting Dianet, Utrecht
dr C.J.A.M. Konings, Catharina Ziekenhuis, Eindhoven
dr J.P. Kooman, Academisch Ziekenhuis Maastricht, Maastricht
mevr. dr M.G. Koopman, Academisch Medisch Centrum, Amsterdam
dr T. Kremer Hovinga, Martini Ziekenhuis, Groningen
dr W.H.M. van Kuijk, VieCuri Medisch Centrum, Venlo
P.B. Leurs, Stichting Oosterscheldeziekenhuizen, Goes
dr L.J.M. Reichert, Ziekenhuis Rijnstate, Arnhem
M.J.M. Schonck, Westfries Gasthuis, Hoorn
dr C.E.H. Siegert, Lucas/Andreas Ziekenhuis, Amsterdam
dr P.J.G. van de Ven, Medisch Centrum Rotterdam Ziekenhuis, locatie Clara
M.G. Vervloet, Vrije Universiteit Medisch Centrum, Amsterdam

10.4 Event committee
This committee will evaluate the following clinical events: cause of death, non fatal myocardial infarction, non fatal CVA, non fatal vascular events. The primary investigators will collect information on mortality and CV events for the event committee (see: data collection). The event committee will consist of persons with different specialisations; a neurologist, a vascular surgeon, an internist, a cardiologist and an epidemiologist (to be determined).

10.5 Data monitoring board
The data monitoring board (DMB) performs statistical analyses of unblinded interim data and formulates recommendations for the steering committee on the continuation of the trial. The DMB may also offer unsolicited recommendations on the continuation of the trial, for example after publication of results of similar trials. Members of the DMB are:
I van der Tweel, biostatistician, Centrum voor Biostatistiek (chair)
prof. dr D.E. Grobbee, clinical epidemiologist, UMC
dr R. Zietse, internist/nephrologist, AZR
prof. dr J. Rauwerda, vascular surgeon, VUMC
prof. dr A.J. Rabelink, internist / vascular medicine, UMC.
The chair of the DMB (I. van der Tweel) will be provided interim datasets at regular intervals (once every 2 months) to perform sequential analyses. When appropriate, given the results from the interim analysis, I. van der Tweel will call for a meeting with the other DMB members or otherwise contact the DMB members.

10.6 Advisory committee
The advisory committee comprises of Dutch leaders in a certain field of medicine. Members of the advisory committee will be consulted when issues arise before, during and after the trial on their field of expertise. Members of the advisory committee who have agreed to participate are:
dr P. Boer, biochemist, UMC
dr M.J. Cramer, cardiologist, UMC
dr O. Kamp, cardiologist, VUmc

prof. dr B. van Rossum, cardiologist, VUmc
prof. dr C.D.A. Stehouwer, internist, VUmc
dr C. Schalkwijk, biochemist, VUmc
dr T. Teerlink, biochemist, VUmc.

11. LIST OF ADDENDA

Addendum I: The treatment of anemia in patients on hemodialysis or online hemodiafiltration.

12. LIST OF ABBREVIATIONS

ACE	angiotensin converting enzyme
ADMA	asymmetric dimethyl arginine
AFB	acetate free biofiltration
AGEs	advanced glycation end products
APR	acute phase response
BI	bioincompatibility
$ß_2$-m	$ß_2$-microglobulin
CFU	colony forming units
CIMT	carotis intima media thickness
CRF	chronic renal failure
CRP	C-reactive protein
CV	cardiovascular
CVD	cardiovascular disease
DGN	Dialyse Groep Nederland
DM	diabetes mellitus
DRA	dialysis related amyloidosis
ECC	extracorporeal circuit
EDTA	European Dialysis Transplant Association
ESRD	end-stage-renal-disease
Hb	hemoglobin
Hcy	homocysteine
HD	hemodialysis
HDF	hemodiafiltration
HDL	high density lipoprotein
HF	hemofiltration
IDL	intermediate density lipoprotein
IMT	intima media thickness
Lp(a)	lipoprotein (a)
LVH	left ventricular hypertrophy
LVMi	left ventricular mass index
MM	middle molecular
MMW	middle molecular weight
NO	nitric oxide
PWV	pulse wave velocity
ROS	reactive oxygen species
sICAM	soluble intercellular adhesion molecule
sVCAM	soluble vascular adhesion molecule
UF	ultrafiltration
vWF	von Willebrand factor

13. REFERENCES

[1] Jungers P, Massy ZA, Nguyen Khoa T, Fumeron C, Labrunie M, Lacour B, Descamps-Latscha B, Man NK. Incidence and risk factors of atherosclerotic cardiovascular accidents in predialysis chronic renal failure patients: a prospective study. Nephrol Dial Transplant 1997; 12: 2597-2602.

[2] Goodman WG, Goldin J, Kuizon BD, Yoon C, Gales B, Sider D, Wang Y, Chung J, Emerick A, Greaser L, Elashoff RM, Salusky IB. Coronary-artery calcification in young adults with end-stage renal disease who are undergoing dialysis. New Engl J Med 2000; 342: 1478-1483

[3] Nubé MJ, Vervloet MG. Pro-atherogene veranderingen ten gevolge van hemodialyse: een kwestie van bioincompatibiliteit? Nederl Tijdschr v Geneesk 2000; 144: 2540-2544

[4] Garg AX, Clark WF, Haynes B, House AA. Moderate renal insufficiency and the risk of cardiovascular mortality: results from the NHANES I. Kidney Int 2002; 61: 1486-1494.

[5] Fried LP, Kronmal RA, Newman AB, et al. Risk factors for 5-year mortality in older adults. The Cardiovascular Health Study. JAMA 1998; 279: 585-592.

[6] Dhondt A, Vanholder R, Van Biesen W, Lameire N. The removal of uremic toxins. Kidney Int 2000; 58 (Suppl 76): S47- S59.

[7] Kielstein JT, Böger TH, Bode-Böger SM, Schäffer J, Barbey M, Koch KM, Frölih JC. Asymmetric dimethylarginine plasma concentrations differ in patients with end-stage renal disease: relationship to treatment method and atherosclerotic disease. J Am Soc Nephrol 1999; 10: 594-600.

[8] Zoccali C, Bode-Boger S, Mallamaci F, Benedetto F, Tripepi G, Malatino L, Cataliotti A, Bellanuova I, Fermo I, Frolich J, Boger R. Plasma concentrations of asymmetrical dimethylarginine and mortality in patients with end-stage renal disease: a prospective study. Lancet 2001; 358: 2113-2117.

[9] Cheung AK, Wu LL, Kablitz C, Leypoldt. Atherogenic lipids and lipoproteins in hemodialysis patients. Am J Kidney Dis 1993; 22: 271-276.

[10] Cressmann MD Heyka RJ, Paganini EP, O'Neil J, Skibinski CI, Hoff HF. Lipoprotein (a) is an independent risk factor for cardiovascular disease in hemodialysis patients. Circulation 1992; 86: 475-482.

[11] Dennis VW, Robinson K. Homocysteinemia and vascular disease in end-stage renal disease. Kidney Int 1996; 50 (Suppl 57): 11-17

[12] Clarke R, Daly L, Robinson K, Naughten E, Cahalane S, Fowler B, Graham I. Hyperhomo-cysteinemia: and independent risk factor for vascular disease. N Engl J Med 1991; 324: 1149-1155

[13] Robinson K, Gupta A, Dennis VW, Arheart K, Chaudhary D, Green R, Vigo P, Mayer EL, Selhub J, Kutner M, Jacobsen D. Hyperhomocysteinemia confers an independent increased risk of atherosclerosis in end-stage renal disease and is closely linked to plasma folate and pyridoxine concentrations. Circulation 1996; 94: 2743-2748

[14] Schnijder G, Fjoffi M, Pin R, flamer Y, Lnage H, Eberli FR, Meier B, Turi ZG, Hess OM. Decreased rate of coronary restenosis after lowering of plasma homocysteine levels. N Engl J Med 2001; 345: 1593-600.

[15] Mimic-Oka J, Simic T, Djukanovic L, Relijc Z, Davicevic Z. Alteration in plasma antioxidant capacity in various degrees of chronic renal failure. Clin Nephrol 1999; 51: 233-241.

[16] Martin-Mateo MC, Sanchez-Portugal M, Iglesias S, de Paula A, Bustamante J. Oxidative stress in chronic renal failure. Renal Failure 1999; 21: 155-167.

[17] Himmelfarb J, Ault KA, Holbroek D, Leeber DA, Hakim RM. Intradialytic granulocyte reactive oxygen species production: a prospective crossover trial. J Am Soc Nephrol 1993; 4: 178-186.

[18] Fiorillo C, Oliviero C, Rizzuti G, Nediani C, Pacini A, Nassi P. Oxidative stress and antioxidant defenses in renal patients receiving regular haemodialysis. Clin Chem Lab Med 1998; 36: 149-153

[19] Singal PK, Khaper N, Palace V, Kumar D. The role of oxidative stress in the genesis of heart disease. Cardiovasc Res. 1998; 40: 426-432.

[20] Steinberg D. Oxidative modification of LDL and atherogenesis. Circulation 1997; 95: 1062-1071.

[21] Annuk M, Zilmer M, Lind L, Linde T, Fellström B. Oxidative stress and endothelial funcion in chronic renal failure. J Am Soc Nephrol 2001; 12: 2747-2752.

[22] Raj DSC, Choudhury D, Welbourne TC, Levi M. Advanced glycation end products: a nephrologist's perspective. Am J Kidney Dis 2000; 35: 365-380

[23] Miyata T, Sugiyama S, Saito A, Kurokawa K. Reactive carbonyl compounds related uremic toxicity ('carbonyl stress'). Kidney Int 2001; 59: 25-31

[24] Makita Z, Yanagisawa K, Kuwajima S, Bucala R, Vlassara H, Koike T. The role of advanced glycosylation end-products in the pathogenesis of atherosclerosis. Nephrol Dial Transplant 1996; 11 (Suppl 5): 31-33.

[25] Brunkhorst R, Kruse M, Ehlerding G, Mahiout A. Advanced glycosylated end-products (AGEs) in non-diabetic patients undergoing dialysis. Clin Nephrol 1996; 46: 265-266.

[26] Miyata T, van Ypersele de Strihou C, Kurokawa K, Baynes JW. Alterations in nonenzymatic biochemistry in uremia: origin and significance of "carbonyl stress" in long-term uremic complications. Kidney Int 1999; 55: 389-399.

[27] Ross R. Atherosclerosis – an inflammaroty disease. N Engl J Med 1999; 340: 115-125.

[28] Danesh J, Collins R, Appleby P, Peto R. Association of fibrinogen, C-reactive protein, albumin or leukocyte count with cornary heart disease. JAMA1998; 279: 1477-1482.

[29] Yeun JY, Levine RA, Mantadilok V, Kaysen GA. C-reactive protein predicts all-cause and cardiovascular mortality in hemodialysis patients. Am J Kidney Dis 2000; 35: 469-476.

[30] Zimmermann J, Herrlinger S, Pruy A, Metzger T, Wanner C. Inflammation enhances cardiovascular risk and mortality in hemodialysis patients. Kidney Int 1999; 55: 648-658.

[31] Docci D, Bilancioni R, Buscaroli A, Baldrati L, Capponcini C, Mengozzi S, Turci F, Feletti C. Elevated serum levels of C-reactive protein in hemodialysis patients. Nephron 1990; 56: 364-367.
[32] Arici M, Walls J. End-stage renal disease, atherosclerosis, and cardiovascular mortality: is C-reactive protein the missing link? Kidney Int 2001; 59: 407-414.
[33] Zoccali C, Benedetto FA, Mallamaci F, Tripepi G, Fermo I, Foca A, Paroni R, Malatino LS. Inflammation is associated with carotid atherosclerosis in dialysis patients. J Hypertens 2000; 18: 1207-1213.
[34] Hak AE, Stehouwer CDA, Bots ML, Polderman KH, Schalkwijk CG, Westendorp ICD, Hofman A, Witteman JCM. Associations of C-reactive protein with measures of obesity, insulin resistance, and subclinical atherosclerosis in healthy, middle-aged women. Arterioscler Thromb Vasc Biol 1999;19:1986-1991.
[35] Guérin AP, Blacher J, Pannier B, Marchais SJ, Safar ME, London GM. Impact of aortic stiffness attenuation on survival of patients in end-stage renal failure. Circulation 2001; 103: 987-992.
[36] Schouten WEM, Grooteman MPC, van Houte AJ, Schoorl M, van Limbeek J, Nubé MJ. Effects of dialyser and dialysate on the acute phase reaction in clinical bicarbonate dialysis. Nephrol Dial Transplant 2000; 15: 379-384.
[37] Van Tellingen A, Grooteman MPC, Schoorl M, Bartels PCM, Schoorl M, van der Ploeg T, ter Wee PM, Nubé MJ. Intercurrent clinical events are predictive of plasma C-reactive protein levels in hemodialysis patients. Kidney Int 202; 62: 632-638.
[38] Panichi V, Migliori M, De Pietro S, Taccola D, Bianchi AM, Horphoth M, Metelli MR, Giovannini L, Tetta C, Palla R. C reactive protein in patients with chronic renal disease. Ren Fail 2001; 23: 551-562.
[39] Stenvinkel P, Heimburger O, Paultre F, Diczfalusy U, Wang T, Berglund L, Jogestrand T. Strong association between malnutrition, inflammation, and atherosclerosis in chronic renal failure. Kidney Int 1999; 55:1899-1911.
[40] Cines DB, Pollak ES, Buck CA, Loscalzo J, Zimmerman GA, McEve RP, Pober JS, Wick TM, Konkle BA, Schwartz BS, Barnathan ES, McCrae KR, Hug BA, Schmidt AM, Stern DM. Endothelial cells in physiology and in the pathofysiology of vascular disorders. Blood 1998; 91: 3527-3561.
[41] Müller MM, Griesmacher A. Markers of endothelial dysfunction. Clin Chem Lab Med 2000; 38: 77-85.
[42] Ridker PM, Hennekens CH, Roitman-Johnson B, Stampfer MJ, Allen J. Plasma concentrations of soluble adhesion molecule 1 and risk of future myocardial infarction in apparently healthy men. Lancet 1998; 351: 88-92.
[43] Yamazaki M, Asakura H, Sato T, Tsugawa Y, Matsumura M, Kawamura Y, Ohka T, Matsuda T. Changes in plasma levels of thrombomodulin during haemodialysis. Blood Coagul Fibrinolysis 1992; 3: 113-117.
[44] Schmidt GW, Moake JL, Rudy CK, Vicks SL, Hamburger RJ. Alterations in hemostatic parameters during hemodialysis with dialyzers of different membrane composition and flow design. Am J Med 1987; 83: 411-418.
[45] Bolton CH, Downs LG, Victory JGG, Dwight JF, Tomson CRV, Mackness MI, Pinkney JH. Endothelial dysfunction in chronic renal failure: roles of lipoprotein oxidation and pro-inflammatory cytokines. Nephrol Dial Transplant 2001; 16: 1189-1197.
[46] Mrowka C, Heintz B, Sieberth HG. Is dialysis membrane type responsible for increased circulating adhesion molecules during chronic hemodialysis. Clin Nephrol 1999; 52: 312-321.
[47] Pannier B, Guérin AP, Marchais J, Metivier F, Safar ME, London GM. Postischemic vasodilation, endothelial activation, and cardiovascular remodeling in end-stage renal disease. Kidney Int 2000; 57: 1091-1099.
[48] Meer IM van der , de Maat MP, Bots ML, Breteler MM, Meijer J, Kiliaan AJ, Hofman A, Witteman JC. Inflammatory mediators and cell adhesion molecules as indicators of severity of atherosclerosis: the Rotterdam Study. Arterioscler Thromb Vasc Biol 2002;22:838-842.
[49] Foley RN, Parfrey PS, Harnett JD, Kent GM, Martin CJ, Murray DC, Barre PE. Clinical and echocardiographic disease in patients starting end-stage renal disease therapy. Kidney Int 1995; 47: 186-192.
[50] Silberberg JS, Barre P, Prichard S, Sniderman AD. Left ventricular hypertrophy: an independent determinant of survival in end stage renal failure. Kidney Int 1989; 36: 286-290.
[51] Zoccali C, Benedetto FA, Mallamaci F, Tripepi G, Giacone G, Cataliotti A, Seminara G, Stancanelli B, Malatino LS. Prognostic impact of the indexation of left ventricular mass in patients undergoing dialysis. J Am Soc Nephrol 2001; 12: 2768-2774.
[52] Koren MJ, Devereux RB, Casale PN, Savage DD, Laragh JH. Relation of left ventricular mass and geometry to morbidity and mortality in uncomplicated essential hypertension. Ann Intern Med 1991; 114: 345-352.
[53] Muiesan S, Salvetti M, Rizzoni D, Castellano T, Donato F, Agabiti-Rosei E. Association of change in left ventricular mass with prognosis during long-term antihypertensive treatment. J Hypertens 1995; 13: 1091-1105.
[54] London GM, Pannier B, Guerin AP, Blacher J, Marchais SJ, Darne B, Metivier F, Adda H, Safar ME. Alterations of left ventricular hypertrophy in and survival of patients receiving hemodialysis: follow-up of an interventional study. J Am Soc Nephrol 2001; 12: 2759-2767.
[55] Cannella G, Paoletti E, Ravera G, Cassottana P, Araghi P, Mulas D, Peloso G, Delfino R, Messa P. Inadequate diagnosis and therapy of arterial hypertension as causes of left ventricular hypertrophy in uremic dialysis patients. Kidney Int 2000; 58: 260-268.
[56] MacDougall IC, Lewis NP, Saunders MJ, Cochlin DL, Davies ME, Hutton RD, Fen KAH, Coles GA, Williams JD. Long-term cardiorespiratory effects of amelioration of renal anemia by erythropoietin. Lancet 1990; 335: 489-493.
[57] Strozecki P, Adamowicz A, Nartowicz E, Odrowaz-Sypniewska G, Wlodarczyk Z, Manitius J. Parathormon, calcium, phosphorus, and left ventricular structure and function in normotensive hemodialysis patients. Ren Fail 2001; 23:115-126.
[58] Hulthe J, Wikstrand J, Emanuelsson H, Wiklund O, de Feyter PJ, Wendelhag I. Atherosclerotic changes in the carotid artery bulb as measured by B-mode ultrasound are associated with the extent of coronary atherosclerosis. Stroke 1997; 28: 1189-1194.

[59] O'Leary DH, Polak JF, Kronmal RA, Manolio TA, Burke GL, Wolfson Jr SK. Carotid-artery intima and media thickness as a risk factor for myocardial infarction and stroke in older adults. New Engl J Med 1999; 340: 14-22.
[60] Iglesias del Sol A, Bots ML, Grobbee DE, Hofman A, Witteman JC. Carotid intima-media thickness at different sites: relation to incident myocardial infarction. The Rotterdam Study. Eur Heart J 2002; 23: 934-940.
[61] Benedetto FA, Mallamaci F, Tripepi G, Zoccali C. Prognostic value of ultrasonographic measurement of carotid intima media thickness in dialysis patients. J Am Soc Nephrol 2001; 12: 2458-2464.
[62] Burdick L, Periti M, Salvaggio A, Bertoli S, Mangiarotti R, Castagnone D, Anguissola G. Relation between carotid artery atherosclerosis and time on dialysis. A non-invasive study in vivo. Clin Nephrol 1994; 42: 121-126.
[63] Kawagishi T, Nishizawa Y, Konishi T, Kawasaki K, Emoto M, Shoju T, Tabata T, Inoue T, Morii H. High-resolution B-mode ultrasonography in evaluation of atherosclerosis in uremia. Kidney Int 1995; 48: 820-826.
[64] Simon A, Gariépy J, Moyse D, Levenson J. Differential effects of nifedipine and co-amilozide on the progression of early carotid wall changes. Circulation 2001;103:2949-2954
[65] Pitt B, Byington RP, Furberg CD, Hunninkhake DB, Mancini GB, Miller ME, Riley W for The PREVENT Investigators. Effect of amlodipine on the progression of atherosclerosis and the occurrence of events. Circulation 2000;102:1503-1510;
[66] Blacher J, Asmar R, Djane S, London G, Safar ME. Aortic pulse wave velocity as a marker of cardiovascular risk in hypertensive patients. Hypertension 1999; 33: 1111-1117.
[67] Laurent S, Boutouyrie P, Asmar R, Gautier I, Laloux B Guize L, Ducimetiere P, Benetos A. Aortic stiffness is an independent predictor of all-cause and cardiovascular mortality in hypertensive patients. Hypertension 2001; 37: 1236-1241.
[68] Blacher J, Guérin AP, Pannier B, Marchais SJ, Safar ME, London GM. Impact of aortic stiffness on survival in end-stage renal disease. Circulation 1999; 99: 2434-2439.
[69] Bots ML, Dijk JM, Oren A, Grobbee DE. Carotid intima-media thickness, arterial stiffness and risk of cardiovascular disease. Current evidence. J Hypertension (in press).
[70] Blacher J, Guérin AP, Pannier B, Marchais SJ, London G. Arterial calcifications, arterial stiffness and cardiovascular risk in end-stage renal disease.Hypertension 2001 ; 38 : 938-942.
[71] Yeun JY, Depner TA. Principles of hemodialysis. Chapter 1, in Dialysis and Transplantation, edited by Owen WF, Pereira BJG, Sayegh MH. Philadelphia, W.B. Saunders Company, 2000. pp 1-31.
[72] Schreiber MJ. Clinical dilemmas in dialysis: managing the hypotensive patient. Setting the stage. Am J Kidney Dis 2001; 38 (Suppl 4): S1-S10.
[73] Grooteman MPC. Bioincompatibility of haemodialysis. Studies on peripheral blood and dialyser eluates. Thesis. Pressofoon Drukkerij, 1996.
[74] Clark WR, Hamburger RJ, Lysaght MJ. Effect of membrane composition and structure on solute removal and biocompatibility in hemodialysis. Kidney Int 1999; 56: 2005-2015.
[75] Ronco C, Brendolan A, Lupi A, Metry G, Levin NW. Effects of a reduced inner diameter of hollow fibers in hemodialyzers. Kidney Int 2000; 58: 809-817.
[76] Mineshima M, Ishimori I, Ishida K, Hoshino T, Kaneko I, Sato Y, Agishi T, Tamamura N, Sakurai H, Masuda T, Hattori H. Effects of internal filtration on the solute removal efficiency of a dialyzer. ASAIO Journal 2000; 46: 456-460.
[77] Clark WR, Macias WL, Molitoris BA, Wang NHL. Plasma protein adsorption to highly permeable hemodialysis membranes. Kidney Int 1995; 48: 481-488.
[78] Ledebo I. On-line hemodiafiltration: technique and therapy. Advances in Renal Replacement Therapy 1999; 6: 195-208.
[79] Pizzarelli F, Maggiore Q. Clinical perspectives of on-line haemodiafiltration. Nephrol Dial Transplant 1998; 13 (Suppl 5): 34-37.
[80] Wizemann V, Külz M, Techert F, Nederlof B. Efficacy of haemodiafiltration. Nephrol Dial Transplant 2001; 16 (Suppl 4): 27-30.
[81] Schinzel R, Münch G, Heidland A, Sebekova K. Advanced glycation end products in end-stage renal disease and their removal. Nephron 2001; 87: 295-303.
[82] Stein G, Franke S, Mahiout A, Schneider S, Sperschneider H, Borst S, Vienken J. Influene of dialysis modalities on serum AGE levels in end-stage renal disease patients. Nephrol Dial Transplant 2001; 16: 999-1008.
[83] van Tellingen A, Grooteman MPC, Barto R, Schalkwijk CG, ter Wee PM, Nubé MJ. Influence of superflux dialyzers on plasma concentrations of low-molecular weight AGE-peptides in chronic hemodialysis patients in the long-term. J Am Soc Nephrol 2001; 12: 417A (abstract).
[84] Gerdemann A, Wagner Z, Solf A, Bahner U, Heidland A, Vienken J, Schinzel R. Plasma levels of advanced glycation end products during haemdodialysis, haemodiafiltration and haemofiltration: potential importance of dialysate quality. Nephrol Dial Transplant 2002; 17: 1045-1049.
[85] Josephson MA, Fellner SK, Dasgupta A. Improved lipid profiles in patients undergoing high-flux hemodialysis. Am J Kidney Dis 1992; 20: 361-366.
[86] Diop ME, Viron B, Bailleul S, Lefevre G, Fessi H, Maury J, Etienne J, Couderc R. Lp(a) is increased in hemodialysis patients according to the type of membrane: a 2-year follow-up study. Clin Nephrol 2000; 54: 210-217.
[87] Seres DS, Strain GW, Hashim SA, Goldberg IJ, Levin NW. Improvement of plasma lipoprotein profiles during high-flux dialysis. J Am Soc Nephrol 1993; 3: 1409-1415.

[88] Goldberg IJ, Kaufman AM, Lavarias VA, Vanni-Reyes T, Levin NW. High flux dialysis membranes improve plasma lipoprotein profiles in patients with end-stage renal disease. Nephrol Dial Transplant 1996; 11 (Suppl 2): 104-107.
[89] Blankestijn PJ, Vos PF, Rabelink AJ, van Rijn HJM, Jansen H, Koomans HA. High flux dialysis membranes improve lipid profile in chronic hemodialysis patients. J Am Soc Nephrol 1995; 5: 1703-1708.
[90] Blankestijn PJ, Joles JA, Koomans HA. Does the modality of haemodialysis treatment affect lipoprotein composition? Nephrol Dial Transplant 1996; 11: 14-16.
[91] Arnadottir M, Berg AL, Hegbrant J, Hultberg B. Influence of haemodialysis on plasma total homocysteine concentration. Nephrol Dial Transplant 1999; 14: 142-146.
[92] House AA, Wells GA, Donnelly JG, Nadler SP, Hébert PC. Randomized trial of high-flux vs low-flux haemodialysis: effects on homocysteine and lipids. Nephrol Dial Transplant 2000; 15: 1029-1034.
[93] Van Tellingen A, Grooteman MP, Bartels PC, Van Limbeek J, Van Guldener C, ter Wee PM, Nubé MJ. Long-term reduction of plasma homocysteine levels by super-flux dialyzers in hemodialysis patients. Kidney Int 2001; 59: 342-347
[94] Schouten WEM, Grooteman MPC, van Houte AJ, Schoorl M, van Limbeek J, Nubé MJ. Effects of dialyser and dialysate on the acute phase reaction in clinical bicarbonate dialysis. Nephrol Dial Transplant 2000; 15: 379-384.
[95] Müller-Steinhardt M, Kock N, Härtel C, Kirchner H, Steinhoff J. Production of monokines in patients under polysulphone haemodiafiltration is influenced by the ultrafiltration flow rate. Nephrol Dial Transplant 2001; 16: 1830-1837.
[96] Panichi V, Migliori M, De Pietro S, Meteli MR, Taccola D, Perez R, Palla R, Rindi P, Cristofani R, Tetta C. Plasma C-reactive protein in hemodialysis patients : a cross-sectional, longitudinal clinical survey. Blood Purif 2000; 18: 30-36.
[97] Schrander-van der Meer AM, ter Wee PM, Kan G, Donker AJM, van Dorp WT. Improved cardiovascular variables during acetate free biofiltration. Clin Nephrol 1999; 51:304-309.
[98] Movilli E, Camerini C, Zein H, D'Avolio G, Sandrini M, Strada A, Maiorca R. A prospective comparison of bicarbonate dialysis, hemodiafiltration, and acetate-free biofiltration in the elderly. Am J Kidney Dis 1996; 27: 541-547.
[99] Altieri P, Sorba G, Bolasco P, Asproni E, Ledebo I, Cossu M, Ferrara R, Ganadu M, Cadinu F, Serra G, Cabiddu G, Sau G, Casu D, Passaghe M, Bolasco F, Pistis R, Ghisu T. Predilution haemofiltration – the Second Sardinian Multicentre Study: comparisons between haemofiltration and haemodialysis during identical Kt/V and session times in a long-term cross-over study. Nephrol Dial Transplant 2001; 16: 1207-1213.
[100] Maggiore Q, Pizzarelli F, Dattolo P, Maggiore U, Cerrai T. Cardiovascular stability during haemodialysis, haemofiltration and haemodiafiltration. Nephrol Dial Transplant 2000; 15 (Suppl 1) : 68-73.
[101] Van Kuijk WHM, Hilion D, Savoiu C, Leunissen KML. Critical role of the extracorporeal blood temperature in the hemodynamic response during hemofiltration. J Am Soc Nephrol 1997;8:949-955.
[102] Maggiore Q, Pizzarelli F, Zoccali C, Sisca S, Nicolo F. Influence of blood temperature on vascular stability during hemodialysis and isolated ultrafiltration. Int J Artif Organs 1985; 8: 175-178.
[103] Van der Sande FM, Kooman JP, Konings CJ, Leunissen KML. Thermal effects and blood pressure response during postdilution hemodiafiltration and hemodialysis: the effect of amount of replacement fluid and dialysate themperature. J Am Soc Nephrol 2001; 1916-1920.
[104] Hakim RM, Held PJ, Stannard DC, Wolfe RA, Port FK, Daugirdas JT, Agodoa L. Effect of the dialysis membrane on mortality of chronic hemodialysis patients. Kidney Int 1996; 50: 566-570.
[105] Leypoldt JK, Cheung AK, Carroll CE, Stannard DC, Pereira BJG, Agodoa LY, Port FK. Effect of dialysis membranes and middle molecule removal on chronic hemodialysis patient survival. Am J Kidney Dis 1999; 33: 349-355.
[106] Hornberger JC, Chernew M, Petersen J, Garber AM. A multivariate analysis of mortality and hospital admissions with high-flux dialysis. J Am Soc Nephrol 1992; 3: 1227-1237.
[107] Bloembergen WE, Hakim RM, Stannard BA, Held PJ, Wolfe RA, Agodoa LYC, Port FK. Relationship of dialysis membrane and cause-specific mortality. Am J Kidney Dis 1999; 33: 1-10.
[108] Locatelli F, Marcelli D, Conte F, Limido A, Malberti F, Spotti D, for the Registro Lombardo Dialisi e Trapianto. Comparison of mortality in ESRD patients on convective and diffusive extracorporeal treatments. Kidney Int 1999; 55: 286-293.
[109] Canaud B, Bosc jy, Leray-Moragues H, Stec F, Argiles A, Leblanc M, Mion C. On-line haemodiafiltration. Safety and efficacy in long-term clinical practice. Nephrol Dial Transplant 2000; 15 (Suppl 1): 60-67.
[110] Locatelli F, Mastrangelo F, Redaelli B, Ronco C, Marcelli D, La Greca G, Orlandini G an the Italian Cooperative Dialysis Study Group. Effects of different membranes an dialysis technologies on patient treatment tolerance an nutritional parameters. Kidney Int 1996; 50: 1293-1302.
[111] Berthoux F, Gellert R, Jones E, Mendel S, Valderrabano F, Briggs D, Carrera F, Cambi V, Saker L. Epidemiology and demography of treated end-stage renal failure in the elderly: from the European Renal Association (ERA-EDTA) registry. Nephrol Dial Transplant 1998; 13 (Suppl 7): 65-68.
[112] Simons PCG, Algra A, Bots ML, Grobbee DE, van der Graaf Y, for the SMART Study Group. Common carotid intima-media thickness and arterial stiffness. Indicators of cardiovascular risk in high-risk patients. The SMART Study (Second Manifestations of ARTerial disease). Circulation 1999; 100: 951-957.
[113] Locatelli F, Fouque D, Heimburger O, Drueke TB, Cannata-Andia JB, Horl WH, Ritz E. Nutritional status in dialysis patients: a European consensus. Nephrol Dial Transplant 2002; 17: 563-572.

[114] Hays RD, Kallich JD, Mapes DL, Coons SJ, CarterWB. Development of the kidney disease quality of life (KDQOL) instrument. Qual Life Res 1994; 3: 329-338.
[115] Korevaar JC, Merkus MP, Jansen MA, Dekker FW, Boeschoten EW, Krediet RT for the NECOSAD-study group. Validation of the KDQOL-SF: a dialysis-targeted health measure. Qual Life Res 2002; 11: 437-447.
[116] Chan CT, Floras JS, Miller JA, Richardson RM, Pierratos A. Regression of left ventricular hypertrophy after conversion to nocturnal hemodialysis. Kidney Int 2002; 61: 2235-2239.
[117] Whitehead J. The design and analysis of sequential clinical trials. Revised 2nd edition. (1997) John Wiley & Sons.
[118] MPS Research Unit (2000). PEST 4: Operating Manual. The University of Reading.
